# ABCA1 transporter promotes the motility of human melanoma cells by modulating their plasma membrane organization

**DOI:** 10.1186/s40659-023-00443-4

**Published:** 2023-06-13

**Authors:** Ambroise Wu, Ewa Mazurkiewicz, Piotr Donizy, Krzysztof Kotowski, Małgorzata Pieniazek, Antonina J. Mazur, Aleksander Czogalla, Tomasz Trombik

**Affiliations:** 1 Department of Cytobiochemistry, Faculty of Biotechnology, University of Wrocław, Joliot-Curie 14a, 50-383 Wrocław, Poland; 2 Department of Cell Pathology, Faculty of Biotechnology, University of Wrocław, Joliot-Curie 14a, 50-383 Wrocław, Poland; 3 Department of Clinical and Experimental Pathology, Wrocław Medical University, Borowska 213, 50-556 Wrocław, Poland; 4 Department of Oncology and Division of Surgical Oncology, Wrocław Medical University, Pl. Hirszfelda 12, 53-413 Wrocław, Poland; 5 Department of Biophysics, Faculty of Biotechnology, University of Wrocław, Joliot-Curie 14a, 50-383 Wrocław, Poland; 6 Present Address: Department of Biochemistry and Molecular Biology, Faculty of Medical Sciences, Medical University of Lublin, Chodzki 1, 20-093 Lublin, Poland

**Keywords:** ABCA1, Cell motility, Cholesterol, Human melanoma, Plasma membrane

## Abstract

**Background:**

Melanoma is one of the most aggressive and deadliest skin tumor. Cholesterol content in melanoma cells is elevated, and a portion of it accumulates into lipid rafts. Therefore, the plasma membrane cholesterol and its lateral organization might be directly linked with tumor development. ATP Binding Cassette A1 (ABCA1) transporter modulates physico-chemical properties of the plasma membrane by modifying cholesterol distribution. Several studies linked the activity of the transporter with a different outcome of tumor progression depending on which type. However, no direct link between human melanoma progression and ABCA1 activity has been reported yet.

**Methods:**

An immunohistochemical study on the ABCA1 level in 110 patients-derived melanoma tumors was performed to investigate the potential association of the transporter with melanoma stage of progression and prognosis. Furthermore, proliferation, migration and invasion assays, extracellular-matrix degradation assay, immunochemistry on proteins involved in migration processes and a combination of biophysical microscopy analysis of the plasma membrane organization of Hs294T human melanoma wild type, control (scrambled), *ABCA1* Knockout (*ABCA1* KO) and ABCA1 chemically inactivated cells were used to study the impact of ABCA1 activity on human melanoma metastasis processes.

**Results:**

The immunohistochemical analysis of clinical samples showed that high level of ABCA1 transporter in human melanoma is associated with a poor prognosis. Depletion or inhibition of ABCA1 impacts invasion capacities of aggressive melanoma cells. Loss of ABCA1 activity partially prevented cellular motility by affecting active focal adhesions formation via blocking clustering of phosphorylated focal adhesion kinases and active integrin β3. Moreover, ABCA1 activity regulated the lateral organization of the plasma membrane in melanoma cells. Disrupting this organization, by increasing the content of cholesterol, also blocked active focal adhesion formation.

**Conclusion:**

Human melanoma cells reorganize their plasma membrane cholesterol content and organization via ABCA1 activity to promote motility processes and aggressiveness potential. Therefore, ABCA1 may contribute to tumor progression and poor prognosis, suggesting ABCA1 to be a potential metastatic marker in melanoma.

**Supplementary Information:**

The online version contains supplementary material available at 10.1186/s40659-023-00443-4.

## Background

Melanoma is a highly aggressive and deadly form of skin tumor due to its large potential of forming metastasis [1]. Cholesterol is one of the critical metabolites as its accumulation in transformed cells is very often required for the tumor survival and development [2, 3]. For the past years, numerous attempts have been made to target cholesterol content at different levels of cellular processes to prevent tumor development [4, 5]. ATP Binding Cassette A1 (ABCA1) transporter is known for its function in reverse cholesterol transport and thus for its ability to modulate cholesterol distribution within the plasma membrane (PM) mostly by promoting its efflux. Fluorescence lifetime imaging microscopy (FLIM) experiments have shown that ABCA1 modifies the composition of lipid rafts by redistributing cholesterol from the nanodomains to other regions within the PM [6]. Moreover, ABCA1 coordinates the formation of nascent high-density lipoprotein (HDL) molecules from the lipid rafts of the PM [7], suggesting the potency of ABCA1 to fine-tune the PM lateral organization [8, 9].

*ABCA1* expression is altered in many types of tumors and the consequences may vary depending on the tumor type. For example, depletion of ABCA1 and loss of cholesterol efflux in prostate cancer led to increased tumor growth [10, 11]. While ABCA1 increased level in colorectal and epithelial ovarian cancer is connected with worse prognoses for patients [12, 13]⁠, the elevated ABCA1 level correlates with reduced tumor growth in colon and oral cancers [14, 15].

Regarding human melanoma, increased expression of genes related to cholesterol synthesis was revealed as a molecular fingerprint of the shorter patients' survival [2]. Simultaneously, disruption of intracellular cholesterol transport in melanoma reduces the viability of melanoma cells [16], suggesting the impact of proteins decreasing cholesterol level on tumor growth. There are limited data regarding the importance of ABC-cassette genes in human melanoma [17, 18] and no direct link between ABCA1 and human melanoma development has been reported to date.

To investigate the impact of ABCA1 on human melanoma progression and level of aggressiveness, we first performed an immunohistochemical study on the ABCA1 level in 110 patients-derived melanoma tumors. We demonstrated that elevated ABCA1 expression is associated with a poor prognosis and higher clinicopathologic advancement. To further study the mechanism underlying these observations, we chose the cell line Hs294T, exhibiting an elevated ABCA1 level and possessing migratory and aggressiveness potential [19]. We demonstrate that the loss of ABCA1 activity alters motility, invasion, and extracellular matrix (ECM) degradation processes of melanoma cells. Moreover, we show that the ABCA1 facilitates focal adhesion proper functioning, by allowing a functional localization of phosphorylated focal adhesion kinase (pFAK^397^) and active integrin β3. Furthermore, by combining two biophysical microscopy techniques, we reveal that the ABCA1 activity modifies the lateral organization, order, and fluidity of the PM of Hs294T cells. Thus, we show that ABCA1 activity promotes the metastatic cells to form functional focal adhesions (FAs) by controlling their PM lateral organization, which enhances their motility.

## Methods

### Antibodies and reagents

Antibody against human ABCA1 (MAB 10005) was purchased from Merck. Antibodies against pFAK^397^ for immunostaining (#44-625G), anti-rabbit IgG HRP (#31466), anti-mouse IgG HRP (#62-6520) anti-rabbit IgG—Alexa Fluor 488 (#A-21206), anti-mouse IgG—Alexa Fluor 647 (#A-3157) were purchased from Invitrogen. Anti-pan F-actin antibodies (AC-40, #A3853) were from Sigma-Aldrich, anti-active integrin β3 (GPIIIa, CD61, EBW106) from Kerafast, anti-α-parvin (D7F9) from Cell Signaling Technology and anti-total FAK (C-903) and pFAK^397^ for Western blot (D20B1) from Santa Cruz. Antibodies against pCREB^133^ (#9191) and pSTAT3^727^ (#9134) were purchased from Cell Signaling Technology. Proteinase and phosphatase inhibitors were purchased from Thermo Fischer Scientific, bovine serum albumin (BSA) and dimethyl sulfoxide (DMSO) from Bioshop, probucol and gelatin from pork skin were from Sigma-Aldrich, cholesterol was from Northern Lipids Inc. and methyl-β-cyclodextrin (MβCD) was from Alfa Aesar. All commonly used biochemical reagents stated below were purchased either from Sigma-Aldrich or Bioshop.

### Immunohistochemistry

Tissue microarrays (TMAs) composed of three 1.5 mm tissue cores from each tumor were automatically constructed (TMA Grand Master, Sysmex, Warsaw, Poland). Immunohistochemical analysis was performed using mouse monoclonal anti-ABCA1 antibody (clone HJ1 (ab66217), dilution 1:200, Abcam) on 4-µm-thick paraffin sections mounted on silanized slides (Agilent DAKO, Santa Clara, CA, USA). The slides underwent automated dewaxing, rehydration, and heat-induced epitope retrieval with EnVision Target Retrieval Solution (Agilent DAKO, Santa Clara, CA, USA) for 30 min at 97 ℃ in PT Link Pre-Treatment Module for Tissue Specimens (DAKO). EnVision Flex HRP Magenta Chromogen (Agilent DAKO, Santa Clara, CA, USA) was utilized as a detection system. Human normal liver was used as a positive control. Negative controls were processed using FLEX Rabbit Negative Control, Ready-to-Use (Agilent DAKO, Santa Clara, CA, USA) in place of the primary antibody. Scoring of ABCA1 immunostaining was performed using the H-score. The score is obtained by the formula: percentage of tumoral cells with weak immunoreactivity × 1 + percentage of tumoral cells with intermediate immunoreactivity × 2 + percentage of tumoral cells with high immunoreactivity × 3, giving a range of 0 to 300. The median H-score (200) was used as a cut-off value for high (H-score > 200) and low ABCA1 (H-score ≤ 200) expression [20].

### Patients

We analyzed 110 cutaneous melanoma patients treated at the Regional Oncology Centre in Opole, Poland, diagnosed in 2005–2010. Patients were enrolled in this study based on the availability of medical documentation and paraffin blocks with primary tumors. This study was reviewed and approved by the ethics committee of the Wroclaw Medical University, Wroclaw, Poland (No 580/2019). The patients did not personally participate in the study, and the results of these investigations did not have any influence on the original treatment of patients because it had already been finished. All investigations were performed in accordance with the Declaration of Helsinki.

Clinical parameters included in this study were age, gender, location of the primary tumor, status of the regional lymph nodes (including the information of sentinel lymph node biopsy (SLNB) procedures), presence of distant metastases, staging according to the 8^th^ version of AJCC, and information concerning disease recurrence. Based on hematoxylin and eosin (H&E) staining of primary tumors, we evaluated histopathologic parameters: Breslow thickness, Clark level, histological type, mitotic rate (number of mitotic figures per 1 mm^2^), presence of ulceration, lymphangioinvasion, microsatellitosis, intensity of tumor-infiltrating lymphocytes (TILs), and microscopic evidence of regression.

### Cells

A375 and Hs294T cell lines were obtained from the American Type Culture Collection (LGC Standards, Lomianki, Poland), WM1341D and WM9 cell lines were from Rockland Immunochemicals, Inc. (Limerick, PA, USA), whereas SK-MEL-28 were bought at the Cell Lines ServiceGmbH (CLS, Eppelheim, Germany). All cell lines were cultured in Dulbecco’s Modified Eagle’s Medium (DMEM, Gibco) supplemented with 10% fetal bovine serum (FBS, Gibco), 100 U/mL penicillin (Gibco), 100 µg/mL streptomycin and 2 mM l-glutamine (Gibco) (complete medium) at 37 °C in a humidified atmosphere containing 5% CO_2_. All cells were subcultured twice per week in Mycoplasma-free conditions.

### CRISPR/Cas9 inactivation of *ABCA1* gene

Hs294T scrambled and *ABCA1* KO cells were generated using the Alt-R CRISPR-Cas9 System (Integrated DNA Technologies) according to the manufacturer’s recommendations. Briefly, Hs294T cells were seeded at 5 × 10^5^ in Opti-MEM (Gibco) in a 6-well plate and transfected with the Alt-R S.p. HiFi Cas9 Nuclease V3 (Integrated DNA Technologies) using Alt-R Lipofectamine^™^ CRISPRMAX^™^ Cas9 Transfection Reagent (Thermo Fischer Scientific). The Alt-R CRISPR-Cas9tracrRNA fused with the Alt-R CRISPR-Cas9 negative control crRNA #1 (Integrated DNA Technologies) was used as a control to generate scrambled cells whereas the Alt-R CRISPR-Cas9tracrRNA fused with the Alt-R CRISPR-Cas9 crRNA (AAGCUGGAGUGACAUGCGACGUUUUAGAGCUAUGCU, Integrated DNA Technologies) was used to generate *ABCA1* KO cells. After transfection, cells were incubated for 48 h at 37 ℃, 5% CO_2_ and CRISPR/Cas9 inactivation was validated by assessing ABCA1 expression level with Western Blot described below. The ABCA1 expression level was routinely controlled in the same way and transfected cells were not subcultured more than 5 times for the experiments.

### Cell treatments

For all experiments except when stated otherwise, Hs294T WT, WMD1341D or WM9 cells were treated either with 0.1% DMSO as vehicle control or with 12.5 µM probucol for 2 h at 37 °C, 5% CO_2_ in complete medium prior to the experiments or cell lysis. For proliferation assay, cell migration assay, gelatin-fluorescein degradation assay and gelatin zymography assay, after probucol or DMSO treatment, cells were kept in a medium with 0.1% DMSO or 12.5 µM probucol during the entirety of the incubation times of the experiments. For invasion assay, Hs294T cells were starved for 24 h in a serum-free medium with 0.1% DMSO or 12.5 µM probucol and seeded onto a Transwell filter in a serum-free medium with 0.1% DMSO or 12.5 µM probucol as described below. To load cells with cholesterol, MβCD:cholesterol (Chol) complexes were produced and used as described in our previous studies [8, 21]. Briefly, scrambled and *ABCA1* KO cells were incubated with Chol (final concentration of 1 mM MβCD and 0.1 mM cholesterol) in a complete medium for 30 min at 37 ℃, 5% CO_2_ prior to the experiments. For DMSO- and probucol-treatment, cells were first treated with 0.1% DMSO or 12.5 µM probucol for 90 min at 37 ℃, 5% CO_2_ then with 0.1% DMSO or 12.5 µM probucol and with Chol (final concentration of 1 mM MβCD and 0.1 mM cholesterol) in complete medium for 30 min at 37 ℃, 5% CO_2_ prior to the experiments.

### Western blot analysis

Cells were harvested, washed three times with Phosphate Buffer Saline 1X (PBS 1X, Gibco) lysed with RIPA buffer (25 mM HEPES, pH 7.4, 150 mM NaCl, 1% NP40, 10 mM MgCl_2_, 1 mM EDTA) supplemented with 2% glycerol and proteinase or proteinase and phosphatase inhibitor cocktails for phosphoproteins for 30 min on ice. After centrifugation for 10 min at 10,000 ×*g*, samples were denatured with two times concentrated loading buffer (8 M urea, 250 mM Tris/HCl pH 6.8, 10% SDS, 20% glycerol, 0.008% bromophenol blue, 100 mM DTT) for ABCA1 level analysis and regular 2 × Laemlli buffer for other proteins, and incubated at 95 °C for 10 min. Protein concentration was measured with ROTI assay and 30 μg of proteins were loaded either on a 5.5% sodium dodecyl sulfate–polyacrylamide gel electrophoresis (SDS-PAGE) for those bigger than 100 kDa and 12% for those smaller than 100 kDa, Then proteins were electro-transferred to polyvinylidene fluoride (PVDF) membranes using the Trans-Blot Turbo transfer system (Bio-Rad) in transfer buffer (48 mM Tris, 39 mM glycine, 0.1% SDS, 10% methanol, pH 9.2). Membranes were then blocked either in 5% skimmed milk in TBS-T (50 mM Tris/HCl pH 7.6, 150 mM NaCl supplemented with 0.05% Tween-20) for detection of ABCA1, FAK and α-parvin or in 5% bovine serum albumin (BSA) in TBS-T for detection of pCREB^133^, pSTAT3^727^ and pFAK^397^ for 1 h at room temperature then incubated overnight at 4 °C with primary antibodies diluted 1:1000 (in 1% skimmed milk in TBS-T for ABCA1, FAK and α-parvin or 1% BSA in TBS-T for pCREB^133^, pSTAT3^727^ and pFAK^397^ ). Excess of primary antibodies was removed by washing the membrane three times in 1% skimmed milk or 1% BSA in TBS-T before incubation with horseradish peroxidase-labeled secondary antibody (1:5000) for 1 h at room temperature. After three washes with TBS-T, the presence of protein was revealed using Western Lightning Plus-ECL (PerkinElmer) on a ChemiDoc System with ImageLab software (Bio-Rad). The densitometric analyses were performed using ImageJ. The bands were standardized to the loading control and then normalized against the mean of protein level in the control group.

### Cholesterol content measurement

The cells were harvested and lysed as described for Western blot analysis. Protein concentration was measured with a ROTI assay. Total and free cellular cholesterol level was determined enzymatically by cholesterol oxidase using the Amplex Red Cholesterol Assay Kit (Thermo Fisher Scientific) according to the manufacturer’s recommendations. For cholesterol content estimation, an equivalent of 5 µg of proteins was used. Samples were mixed with Amplex Red reagent/HRP/cholesterol oxidase/cholesterol esterase working solution for total cholesterol Amplex Red reagent/HRP/cholesterol oxidase working solution for free cholesterol and incubated for 30 min at 37 °C in darkness. Fluorescence was measured using excitation at 560 nm and emission detection at 590 nm with a Cary Eclipse fluorescence microplate reader (Agilent Technologies) for melanoma cell lines or with a GloMax Discover Microplate Reader (Promega) for Hs294T scrambled, *ABCA1* KO, DMSO- and probucol-treated cells. The background was subtracted from the final readout. The cholesterol concentration was established using a standard curve. The final cholesterol content was calculated in ng of cholesterol per µg of protein. Esterified cholesterol content was determined by subtracting the free cholesterol content from the total cholesterol content.

### Proliferation

Proliferation assays were performed using a 5 Bromodeoxyuridine (BrdU) cell proliferation assay kit (BioVision) according to the manufacturer. Briefly, Hs294T WT, scrambled, and *ABCA1* KO cells were seeded at 3 × 10^3^ in 96 wells plate and incubated at 37 °C, 5% CO_2_ for 72 h. Then non-treated and treated cells were incubated with BrdU solution at 37 °C for 4 h. After removal of BrdU solution, cells were incubated with a Fixing/Denaturing Solution at room temperature for 30 min. BrdU Detection Antibody solution was added to each well and the plate was incubated at room temperature for 1 h with gentle shaking. After two washes with the Wash buffer, anti-mouse HRP-linked Antibody Solution was added for 1 h at room temperature. After three washes, the substrate was added to the wells for 30 min and then the enzymatic reaction was stopped with the Stop solution. The absorbance of wells was finally measured at 450 nm with a μQuant plate reader (Bio Tek Instruments, Inc.). The BrdU incorporation is presented as a relative BrdU incorporation factor (fold).

### Cell migration assay

2D spontaneous migration was monitored using IncuCyte ZOOM System (Essen BioScience) as described earlier [22]. Hs29T scrambled, *ABCA1* KO and Hs294T, WM1341D or WM9 DMSO- and probucol-treated cells were seeded at 1 × 10^3^ cells per well into 96-well IncuCyte ImageLock plates (Essen BioScience) and image sets have been collected every 2 h for 72 h. The single-cell distance has been analyzed using the Manual Tracking plug-in for ImageJ (F. Cordelieres, Institute Curie, Paris, France).

### Invasion assay

The cell invasion assay was performed using Transwell filters (BD Bioscience) coated with Matrigel (BD Bioscience) at a final concentration of 1 mg/mL diluted in a serum-free medium as described earlier [22]. Twenty-four hours later, after serum starvation, Hs294T scrambled, *ABCA1* KO and Hs294T, WM1341D and WM9 DMSO- and probucol-treated cells were seeded onto a Transwell filter with a Matrigel layer in 500 µL of serum-free medium. As a chemoattractant, placed in a lower compartment, a medium containing only 20% FBS was used. Upon 24 h incubation at 37 °C, cells remaining on the upper side of the filter were removed together with Matrigel. The cells on the lower side of the filter were fixed with 4% formaldehyde solution for 20 min at room temperature, stained with Hoechst 33342 (1:2000, Thermo Fisher Scientific) and counted under a fluorescent microscope (Olympus). The cells with invasion ability are presented as a relative invasion factor (fold), with 1 taken as the number of cells invading under control conditions.

### ECM degradation assay

Fluorescein-gelatin degradation assays were performed as described previously [23]. Briefly, Hs29T scrambled, *ABCA1* KO, DMSO- and probucol-treated cells were seeded at 2 × 10^4^ cells on fluorescein-labeled gelatin-coated glass coverslips and incubated for 12 h at 37 °C, 5% CO_2_ in complete medium. Cells were then washed three times with PBS 1X, fixed 4% formaldehyde solution for 20 min at room temperature, washed once again three times with PBS 1X and labeled with Hoechst 33342 (1:1000) and Alexa Fluor 568-labeled phalloidin (1:100, Thermo Fischer Scientific) to stain nuclei and F-actin, respectively. After three final washes with PBS 1X, and once with deionized H_2_O, coverslips were mounted on slides using Dako Mounting Medium and cells were imaged using a 63X oil immersion objective on a LEICA SP8 confocal microscope (LEICA) with LAS X software. The numbers of cells digesting the fluorescein-gelatin and of the invadopodia formed were estimated manually by using the ImageJ software and expressed as percentages.

### Gelatin zymography assay

The assay was performed as described previously [24]. Briefly, Hs294T WT, scrambled and *ABCA1* KO cells were cultured in 75 cm^2^ flasks in a complete medium at 37 °C, 5% CO_2_, until reaching 70% of confluence. Cells were then washed thoroughly and incubated with serum-free medium and treated with probucol or vehicle (DMSO) 72 h at 37 °C, 5% CO_2_. Media were then collected, centrifuged for 20 min at 7000 ×*g* at 4 °C and concentrated with an Amicon Ultra centrifugal filter with a 10 kDa cutoff (Merck). Then, 5 µg of the concentrated media were mixed to a final ratio of 1:1 with 2 × nonreducing loading buffer (10 mM Tris pH 6.8, 1% SDS, 10% glycerol, 0.03% bromophenol blue), incubated for 20 min at 37 °C and loaded into 10% SDS-PAGE with 2.65 mg/ml of gelatin in H_2_O. After the electrophoresis, gels were washed with a Washing buffer (50 mM Tris pH 7.5, 150 mM NaCl, 10 mM CaCl_2_, 2.5% Triton X-100) twice for 30 min and activated in the incubation buffer (50 mM Tris pH 7.5, 150 mM NaCl, 10 mM CaCl_2_) for 16 h at 37 °C. Gels were then stained with the Staining solution (0.5% Coomassie Brilliant Blue R-250 (Merck), 30% of ethanol, and 10% of acetic acid) for 30 min at room temperature with gentle agitation. Gels were finally incubated in the Destaining buffer (30% of ethanol and 10% of acetic solution in H_2_O) until clear bands representing the gelatinase activity were visible and pictures were taken with a ChemiDoc System (BioRad). In parallel, 5 µg of the concentrated media were loaded in 10% SDS-PAGE and gels were stained with the staining solution to assess the total protein content and used as a loading control. The densitometric analyses were performed as described previously. The ratio of active MMP compared to pro-MMP was determined and expressed as percentages.

### Immunostaining and confocal microscopy

Two days prior to the experiments, Hs294T WT, scrambled and *ABCA1* KO cells were seeded at 1 × 10^4^ on glass coverslips and incubated at 37 °C, 5% CO_2_ in a complete medium. Then, non-treated and treated cells were washed three times and fixed either with ice-cold methanol for 10 min (for pFAK^397^ combined with either total F-actin or active integrin β3 staining) or with formaldehyde solution for 20 min at room temperature, washed three times in PBS 1X and permeabilized with PBS 1X/0.1% Triton X-100 (for α-parvin and active integrin β3 staining). After three other washes, cells were blocked with PBS 1X/BSA 1% for 1 h at room temperature. Cells were then labeled with antibodies against pFAK^397^ (1:200), pan actin (1:100), active integrin β3 (1:100), or α-parvin (1:200) for 1 h at room temperature in PBS 1X/BSA 0.1%. Cells were washed once again three times and labeled with secondary antibodies conjugated with Alexa Fluor-488 (pFAK^397^ and α-parvin) or Alexa Fluor-647 (total F-actin and active integrin β3 staining) for 1 h at room temperature covered from the light. Cells were finally washed three times with PBS 1X and once with deionized H_2_O and coverslips were mounted on as previously described. Cells were imaged using a 63X oil immersion objective on a STELLARIS confocal microscope with LAS X software (Leica). The number of cells enriched in pFAK^397^ FAs and with active integrin β3 clusters enriched in pFAK^397^ were determined manually with the ImageJ software and expressed as percentages. The active integrin β3 cluster numbers and sizes were determined with the ImageJ software by uniformly adjusting the threshold (min = 40 and max = 255) to remove the background signal and using the ‘analyze particles’ function while limiting the size between 0.50 and 5 µm^2^ to remove non clustered structures and clustered that were not resolutely separated enough. The area of cell spreading was also measured using the ImageJ software.

### svFCS measurments

Two days prior to the experiments, Hs294T WT, scrambled and *ABCA1* KO cells were seeded at 1 × 10^4^ in cells 8-wells Lab-Tek Chamber Slides (Nunc) and incubated at 37 °C, 5% CO_2_ in a complete medium. Immediately before measurements, non-treated and treated cells were washed three times in HBSS (Gibco) supplemented with 10 mM HEPES, pH 7.4 (Gibco). Then cells were labeled with the fluorescent sphingomyelin lipid analog (Bodipy-SM, Thermo Fisher Scientific) at 0.075 µM lipid/BSA complex in HBSS-HEPES for 10 min at room temperature in the dark. After incubation, cells were washed again with HBSS-HEPES. The svFCS measurements were performed using a custom-made svFCS apparatus based on Axiovert 200 M microscope (Zeiss) and C-Apochromat 40 ×, 1.2 NA with an excitation of 488 nm using a laser beam focused, according to the protocol described by Mailfert *et* al. [25]. Briefly, the waist size (ω^2^) was calibrated using 2 nM Rhodamine 6G solution and 488 nm laser beam illumination at the intensity of 330 μW. For living cells analysis, the 488 nm laser beam was adjusted to 2–4 μW and the signal was collected via a series of 20 runs lasting for 5 s each. The measurements were carried out on 10 to 20 individual cells and the obtained data were analyzed by the IGOR Pro software (WaveMetrics). The collected autocorrelation functions were fitted with a 2D lateral diffusion model and the mean diffusion time τd was calculated. Four waists were analyzed in order to construct a single diffusion law.

### FLIM analysis

Two days prior to the experiments, Hs294T WT, scrambled and *ABCA1* KO cells were seeded at 1 × 10^4^ in 8-wells Lab-Tek Chamber Slides and incubated at 37 °C, 5% CO_2_ in a complete medium. Immediately before measurements, non-treated and treated cells were washed three times in HBSS-HEPES and then incubated with 4 μM di-4 ANEPPDHQ (Di-4, Life Technologies) for 15 min at 37 °C. FLIM measurements were performed at 37 °C using an LSM 510 META microscope (Zeiss) equipped with FLIM/FCS module from PicoQuant. Samples were excited at 470 nm pulsed laser and imaged with a 40 × C-Apochromat W objective (NA 1.2, Zeiss). Fluorescence was collected through a 500 nm long-pass filter. Acquisition time was adjusted to collect at least 1000 photons per pixel. Laser power was adjusted to achieve a photon collection not exceeding 900 photons/s. Data acquisition and processing were conducted using the SymPhoTime software (PicoQuant). Each pixel in the image was pseudo-colored according to the average fluorescence lifetime. Images were analyzed using SymphoTime software (PicoQuant). To analyze only the fluorescence of the cell plasma membranes, background and signal from inside the cells were removed. Di-4 fluorescence lifetimes were obtained from the average of the lifetime with maximum fluorescence intensity of each cell condition. Di-4 lifetime intensity distributions were obtained by segmenting lifetimes in two-lifetime segments: from 3 to 4 ns ([3, 4]) and from 4 to 5 ns ([4, 5]). The sum of fluorescence intensity of each segment was divided by the total sum of fluorescence intensity of both segments and is reported as a percentage.

### Statistics

The statistical analysis of patients-derived tumor tissue model results was conducted in R language (version 3.6.2, https://www.r-project.org) with the use of ‘RStudio’ tool (1.2.5033, [26]) and packages; ‘survminer’ [27] ‘ggplot2’ [28], and ‘ggpubr’ [29]. Statistical significance between the ABCA1 H-score means within groups divided by different clinical and pathologic parameters was calculated by Wilcoxon two sample tests (for two groups) or ANOVA (for three or more groups). To perform correlations, we divided all patient cases into two groups based on ABCA1 H-score—‘low’ and ‘high’, respectively with the H-score ≤ 200 (below or equal median value and > 200 (above median value. The correlations between ABCA1 H-score and clinical and pathologic variables were obtained by use of chi-square test (for categorical variables), or Fisher exact test (for binary variables), or Wilcoxon two sample tests (for continuous variables). The Kaplan–Meier curves for CSOS and DFS with log-rank significance test were prepared to analyze the ABCA1 H-score’s impact on patients’ survival. The Shapiro–Wilk test was used to evaluate the normal distribution of continuous data.

The statistical analyses of cell line model results were performed using Origin 2018. The statistical significance of differences was assessed either by one-way ANOVA with Sidak’s multiple comparisons or by the unpaired Student t-test. For all tests, the significance level (alpha, α) was set to 0.05. Significant differences are indicated in individual graphs with p values as follows: *p ≤ 0.05, **p ≤ 0.01, ***p ≤ 0.001, ****p ≤ 0.0001. If no differences were observed, ns (non-significant) is noted in the graphs. For each result, an appropriate description of the analysis appears in the figure legend.

## Results

### Expression of ABCA1 in cutaneous melanoma patients and analysis of correlations between ABCA1 expression and clinicopathologic parameters

**Fig. 1:**
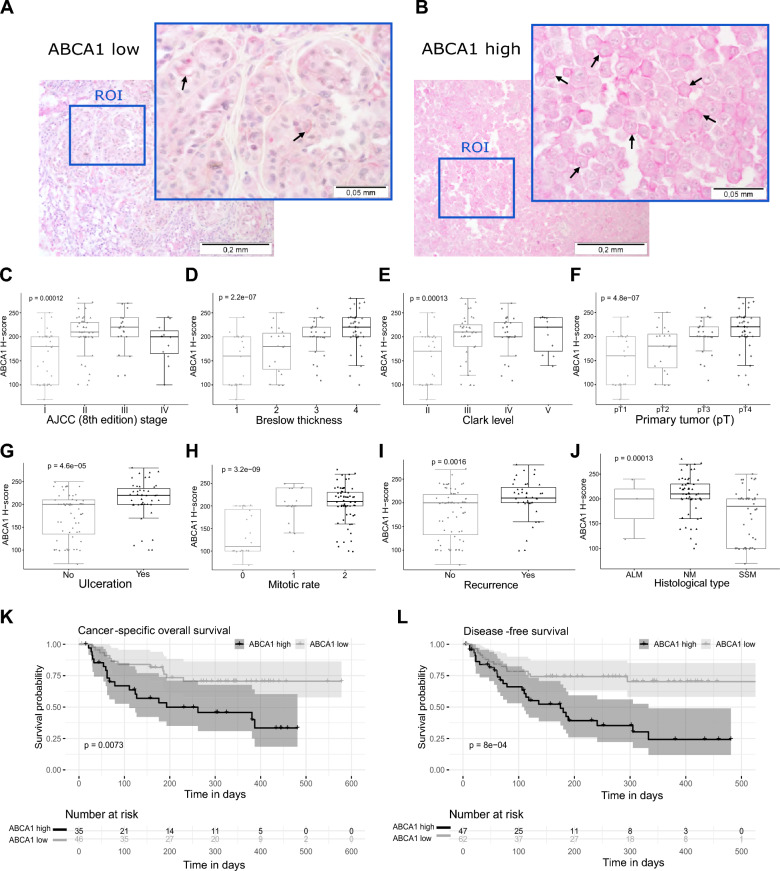
Clinicopathologic analysis of ABCA1 expression in cutaneous melanoma patients.** A**, **B** Representative images of ABCA1 immunoreactivity in cutaneous melanoma cells (**A**; low level of expression 200 × and 600 ×, **B**; high expression 200 × and 600 ×) Black arrows indicate membranous localization of ABCA1. Scale bars represent 0.2 mm (200 ×) or 0.05 mm (600 ×). *ROI* region of interest. **C**, **J** Box-plot comparison of H-score means between the groups divided by different clinical and histological parameters. ANOVA or Wilcoxon two sample tests were used for statistical analyses. Mitotic rates were defined as: 0—no mitosis (0 mitoses/mm^2^), 1—low mitogenic potential (1–2 mitoses/mm^2^) and 3—highly mitogenic tumors (≥ 3 mitoses/mm^2^) pT: primary tumour, ALM: acral lentiginous melanoma, NM: nodular melanoma, SSM: superficial spreading melanoma. **K**, **L** Kaplan–Meier analysis of the impact of ABCA1 on long-term survival of cutaneous melanoma patients. Overexpression of ABCA1 in melanoma cells significantly correlated with shorter cancer-specific overall survival (**K**) and shorter disease-free survival (**L**). The log-rank test was used for statistical analyses

We first evaluated ABCA1 expression by immunohistochemistry performed on tissue microarrays generated from 110 primary cutaneous melanomas (archival formalin-fixed, paraffin-embedded specimens). ABCA1 immunoreactivity was measured with the H-score method. ABCA1 H-scores ranged from 70 to 280, and the mean H-score value was 190 (± 50.3), median: 200. In all positive cases, we observed predominantly cytoplasmic-membranous ABCA1 localization (Fig. 1A and B). For the statistical analysis, we divided the study group into two subgroups: (1) low ABCA1 expression (defined as an H-score ≤ 200) and (2) high ABCA1 expression (defined as an H-score > 200). Low ABCA1 immunoreactivity was observed in 63 patients (57.3%), and ABCA1 overexpression was observed in 47 patients (42.3%).

Further analysis of the tissue microarrays revealed that high expression of ABCA1 significantly correlated with more advanced tumors according to American Joint Committee on Cancer (AJCC) staging, the Breslow thickness and Clark level of invasion (p < 0.001, p = 0.001, and p < 0.001, respectively) (Fig. 1C–F, Additional file 1). Enhanced expression of ABCA1 was also correlated with the presence of ulceration (p < 0.001, Fig. 1G), one of the most important negative histopathologic prognostic factors in cutaneous melanoma patients, and high mitotic activity of primary tumors (p < 0.001, Fig. 1H). Moreover, reduced immunoreactivity of ABCA1 in melanoma cells was significantly associated with low risk of recurrence (p = 0.003, Fig. 1I). Increased ABCA1 reactivity in tumoral cells is a potential marker of nodular type of cutaneous melanoma. We revealed a significant association between high ABCA1 expression and this prognostically unfavorable subtype of cutaneous melanoma (p < 0.001) (Fig. 1J, Additional file 1). Finally, survival analysis showed important impact of ABCA1 on long-term survival of cutaneous melanoma patients. Overexpression of ABCA1 in melanoma cells significantly correlated with shorter cancer-specific overall survival and shorter disease-free survival (p = 0.007 and p < 0.001, respectively, Fig. 1K and L). Altogether, the results suggest that high expression of ABCA1 is linked with highly aggressive clinical behavior of human melanoma.

### The role of ABCA1 activity in cholesterol homeostasis of melanoma cells

To further understand the impact of ABCA1 activity on human melanoma progression, we analyzed the protein level of ABCA1 in five different melanoma cell lines using the Western blot technique. Three cell lines, WM1341D, SK-Mel-28 and A375 were derived from primary tumor sites whereas two cell types, WM9 and Hs294T, were isolated from metastatic sites. A high level of ABCA1 was observed for WM1341D, WM9 and Hs294T, whereas SK-Mel-28 and A375 exhibited low levels of that protein (Additional file 2: Fig. S1A and B). We also observed that the total cellular cholesterol content was inversely proportional to the level of ABCA1 (Additional file 2: Fig. S1C). This result suggests that the transporter was active and exporting cholesterol. We decided to select the Hs294T cell line to study the impact of ABCA1 activity on melanoma cells’ metastatic abilities for the following reasons: a high level of ABCA1 combined with the lowest amount of cholesterol and, as described in a previous study, the highest metastatic potential in comparison to the other four cell lines [19].

**Fig. 2:**
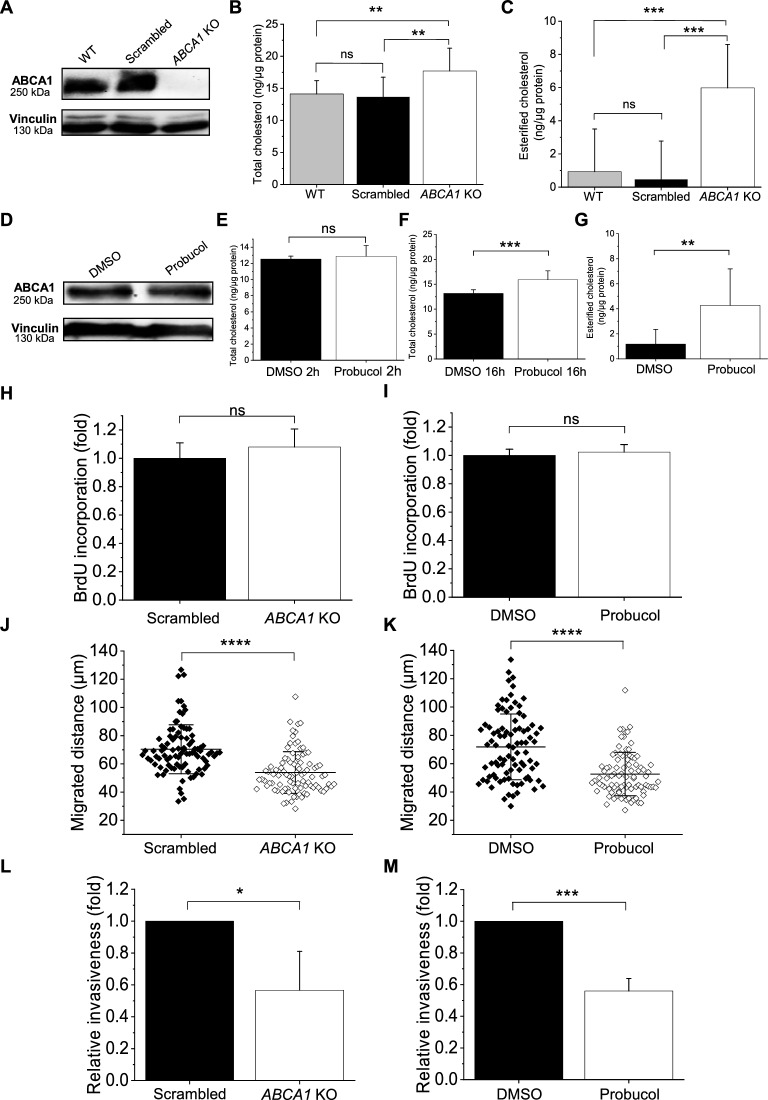
Loss and inhibition of ABCA1 activity impacts migration and invasion abilities of Hs294T cells but not their proliferation. **A** Representative immunoblots of ABCA1 level of WT and CRISPR/Cas9 generated scrambled and *ABCA1* KO cells. Vinculin was analyzed as a loading control (n = 3). **B** Total cholesterol quantitative measurement of WT, scrambled and *ABCA1* KO cells. (n = 9 from three independent experiments, one-way ANOVA with Sidak’s multiple comparisons test). **C** Esterified cholesterol quantitative measurement of WT, scrambled and *ABCA1* KO cells (n = 9 from three independent experiments, one-way ANOVA with Sidak’s multiple comparisons test. **D** Representative immunoblots of ABCA1 level of Hs294T WT cells treated either with DMSO (vehicle) or 12.5 μM probucol for 2 h. Vinculin was analyzed as a loading control (n = 3). **E** Total cholesterol quantitative measurement assay of DMSO- or 12.5 μM probucol-treated cells for 2 h (n = 9 from three independent experiments, unpaired Student t-test). **F** Total cholesterol quantitative measurement of DMSO- or 12.5 μM probucol-treated cells for 16 h (n = 9 from three independent experiments, unpaired Student t-test). **G** Esterified cholesterol quantitative measurement of DMSO- or 12.5 μM probucol-treated cells for 16 h (n = 9 from three independent experiments, unpaired Student t-test). **H**, **I** Quantification of relative proliferation potential after BrdU incorporation assay of scrambled and ABCA1 KO cells (**H**) or of DMSO- or probucol-treated cells (**I**) (n = 9 from three independent experiments, unpaired Student t-test). **J**, **K** Calculated covered distances of scrambled and ABCA1 KO (**J**) or DMSO- and probucol-treated cells (**K**) after 72 h (n = 90 from three independent experiments, unpaired Student t-test). **L**, **M** Relative invasiveness of scrambled and ABCA1 KO cells expressed as fold change of scrambled (**L**) or DMSO- and probucol-treated cells expressed as fold change of DMSO-treated cells (**M**) after 24 h (n = 9 from three independent experiments, unpaired Student t-test). Data are mean ± SD. *ns* non-significant, *p ≤ 0.05, ***p ≤ 0.001 and ****p ≤ 0.0001

We generated *ABCA1* knockout Hs294T cells (*ABCA1* KO) and the scrambled cells as control using the CRISPR/Cas9 technique. Immunoblot analysis confirmed the absence of ABCA1 in the *ABCA1* KO cells (Fig. 2A). Afterward, we assessed the total cholesterol content after the loss of ABCA1 protein level and we observed that in *ABCA1* KO cells the total cholesterol content was higher than in wild-type (WT) and scrambled cells, 17.7, 14.1 and 13.7 ng/μg of total protein, respectively (Fig. 2B). These results clearly show that cholesterol was accumulated in the cells deprived of ABCA1. High accumulation of cholesterol leads to the deregulation of cellular metabolism and is toxic to cells [30-32]. To mitigate the deleterious effect, cells can metabolize the accumulated cholesterol into esterified cholesterol that can be harmlessly stored within cells [33]. To verify if such a process was happening after *ABCA1* depletion, we quantified the amount of esterified cholesterol (Fig. 2C). Around six times higher content of esterified cholesterol in *ABCA1* KO cells compared to WT and scrambled cells indicated that in the absence of ABCA1-mediated cholesterol export, the cells esterify the accumulated cholesterol in order to maintain proper cellular functions.

To ascertain that further changes in cellular functions were indeed made strictly by the loss of *ABCA1* expression, we conducted in parallel similar set of experiments using WT cells treated with probucol, a specific inhibitor of the ABCA1 activity the effectiveness of which we already prove in our previous study [21]. We first confirmed that the level of ABCA1 protein was not altered between Hs294T cells treated either with DMSO or with 12.5 µM probucol for 2 h (Fig. 2D) and then measured the cholesterol content. However, after only 2 h of treatment with probucol, we did not see differences between DMSO- and probucol-treated cells (Fig. 2E). The explanation may lay in the fact, that the synthesis of cholesterol is a complex process with thirty steps that requires time [32]. Hence, we incubated the cells with probucol for 16 h and we noted that they accumulated cholesterol to a higher extent than DMSO-treated ones, but similarly to *ABCA1* KO cells. This shows that the ABCA1 activity was indeed inhibited by the chemical compound (Fig. 2F). Moreover, the amount of esterified cholesterol in the probucol-treated cells was also higher than in DMSO-treated cells suggesting that the cells with inhibition of ABCA1 activity employed the same homeostasis mechanism upon cholesterol accumulation as the *ABCA1* KO cells (Fig. 2G).

### ABCA1 depletion and inactivation alter physiological processes in Hs294T melanoma cells

We first assessed the proliferation potential of the studied cells mentioned above with the BrdU incorporation assay. However, no difference in proliferation was observed for all conditions (Fig. 2H and I). We then monitored the ability of *ABCA1* KO and probucol-treated cells to migrate spontaneously. Both *ABCA1* KO cells and probucol-treated cells covered significantly shorter distances over 72 h, with an average of 53.4 and 52.6 µm, respectively, compared to scrambled and DMSO-treated cells, with an average of 70.4 and 71.8 µm, respectively (Fig. 2J and K). Finally, we analyzed the invasion potential of studied cells using Transwell^™^ filters coated with Matrigel^™^ that mimics the ECM [34]. Both *ABCA1* KO and probucol-treated cells exhibited a reduced capacity for invading the ECM and migrating to the other side of the filter, which was reflected by a loss of more than 40% of invading cells when compared to scrambled and DMSO-treated cells (Fig. 2L and M). Additionally, in the two other melanoma cell lines highly expressing ABCA1, namely WM1341D and WM9 (Additional file 2: Fig. S1), we also observed a significant diminution of the total migrated distance (Additional file 2: Fig. S2A, B) as well as a loss of invasion potential (Additional file 2: Fig. S2C, D) upon probucol treatment. All these outcomes suggest that the ABCA1 activity is involved in the motility of melanoma cells, impacting their signalization, migration and invasion abilities but not proliferation in vitro.

### ABCA1 activity is important for extracellular matrix degradation

**Fig. 3:**
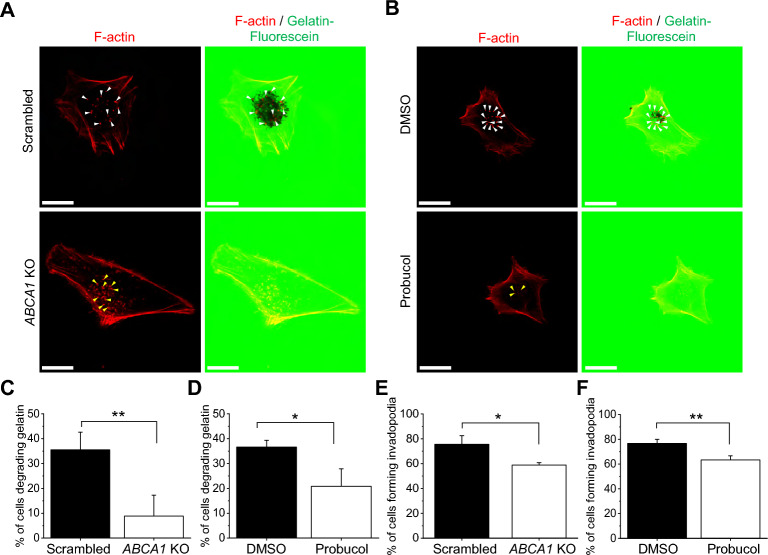
Loss of the ABCA1 activity reduces the ability of cells to form active invadopodia. **A**, **B** Representative confocal microscopy images of scrambled and *ABCA1* KO cells (**A**) or DMSO- and probucol-treated cells (**B**) seeded on gelatin-fluorescein (green) coverslips and F-actin stained with phalloidin-Alexa Fluor 568 (red). White arrowheads point at gelatin degrading invadopodia and yellow arrowheads non-active invadopodia. Scale bars represent 20 µm. **C**, **D** Quantification of cell number degrading gelatin of scrambled and *ABCA1* KO cells (**C**) or DMSO- and probucol-treated cells (**D**) expressed as % of cells degrading gelatin from the total number of cells analyzed (n = 30 from three independent experiments, unpaired Student t-test). **E**, **F** Quantification of cells number forming invadopodia of scrambled and *ABCA1* KO cells (**E**) or DMSO-and probucol-treated cells (**F**) expressed as % of invadopodia forming cells from the total number of cells analyzed (n = 30 from three independent experiments unpaired Student t-test). Data are mean ± SD. *p ≤ 0.05 and **p ≤ 0.01

Since we observed that both migration and invasion processes were affected by the loss of ABCA1 activity, we wanted to know whether the lowering of invasion potential was only due to the loss of the motility or if the ECM degradation ability of the cells was also altered. To do so, we seeded the cells on gelatin conjugated with fluorescein and analyzed the gelatin-fluorescein digested area with a confocal microscope [23]. Additionally, we stained the cells with fluorescently labeled phalloidin to detect filamentous (F-) actin, which forms the core of an invadopodium. We observed some areas with a loss of fluorescein fluorescence under scrambled and DMSO-treated cells, in which distinct F-actin spots were colocalizing, indicating gelatin degradation by invadopodia. On the other hand, *ABCA1* KO and probucol-treated cells were less effective in digestion, even though F-actin spots were still visible (Fig. 3A and B). Quantification confirmed that 35.5% and 36.6% of scrambled and DMSO-treated cells with functional ABCA1 transporter were able to degrade gelatin, respectively. The corresponding proportion of *ABCA1* KO and probucol-treated cells decreased to 8.8% and 20.8%, respectively, indicating an influence of ABCA1 activity on gelatin degradation (Fig. 3C and D). Consistently, 75.5% of scrambled cells and 76.6% of DMSO-treated cells formed invadopodia, compared to 58.8% of *ABCA1* KO cells and 63.3% of probucol-treated cells (Fig. 3E and F), which suggests an involvement of ABCA1 in invadopodia formation in Hs294T cells.

Degradation of the ECM by tumor cells depends on several secreted proteinases, such as MMPs that are proficient in digesting different ECM substrates [35]. Thus, we investigated whether ABCA1 could impact the activity of MMP-2 and MMP-9, two MMPs known to degrade gelatin matrix [36], by performing a gelatin zymography test with a conditioned medium [24] on the studied cells. However, we did not observe significant differences in the activity of the two MMPs either between *ABCA1* KO and scrambled cells or between probucol- and DMSO-treated cells (Additional file 2: Fig. S3). These results suggest that the ABCA1 activity is important for the formation of active invadopodia involved in the ECM degradation, but the level of active MMPs in the conditioned medium remains unaltered in response to the changed ABCA1 activity.

### ABCA1 activity impacts focal adhesion formation

**Fig. 4:**
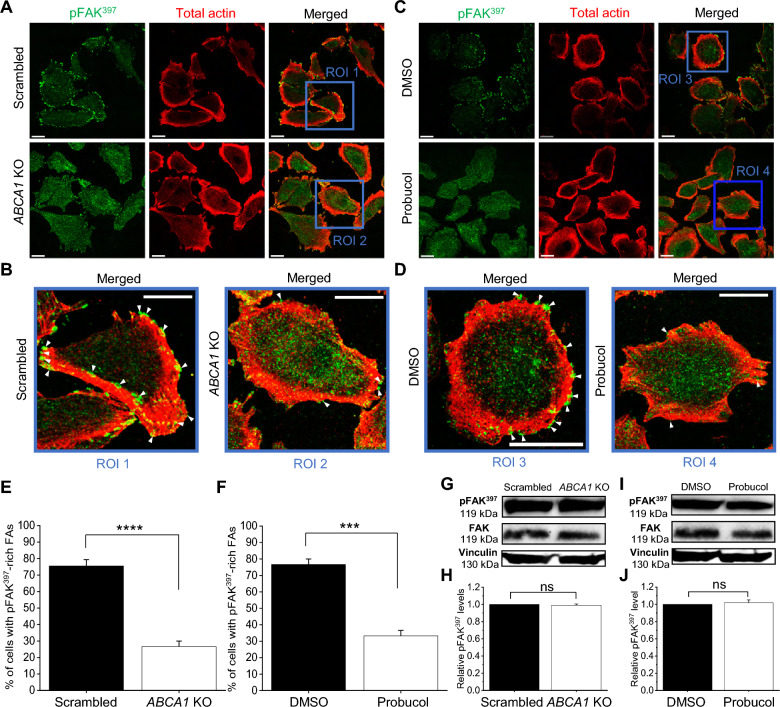
Stable recruitment of active form of FAK in FAs is dependent on ABCA1 activity.** A** and **C** Representative confocal images of scrambled and *ABCA1* KO cells (**A**) or DMSO- and probucol-treated cells (**C**) stained with an antibody recognizing pFAK^397^ followed by secondary antibody conjugated with Alexa Fluor 488 (green) and antibody against total actin followed by secondary antibody conjugated with Alexa Fluor 647 (red) after fixation. Scale bars represent 20 µm. **B** and **D** Magnified view of scrambled (ROI1) and *ABCA1* KO (ROI2) cells (**B**) from (**A**) or of DMSO- (ROI3) and probucol-treated (ROI4) cells (**D**) from (**C**). White arrowheads point at enriched in pFAK^397^ FAs. Scale bars represent 20 µm. **E**, **F** Quantification of cells number exhibiting enriched pFAK^397^ FAs in scrambled and *ABCA1* KO cells (**E**) or DMSO- and probucol-treated cells (**F**) expressed as % of cells with enriched pFAK^397^ FAs from the total number of cells analyzed (n = 30 from three independent experiments, unpaired Student t-test). **G** and **I** Representative immunoblots of pFAK^397^ and total FAK levels in scrambled and *ABCA1* KO cells (**G**) or DMSO- and probucol-treated cells (**I**). Vinculin was used as a loading control. **H** and **J** Quantification of pFAK^397^ relative levels of scrambled and *ABCA1* KO cells (**H**) from (**G**) or of DMSO- and probucol-treated cells (**J**) from (**I**). (n = 3 independent experiments, unpaired Student t-test). *ROI* region of interest. Data are mean ± SD. *ns* non-significant and ****p ≤ 0.0001

As we observed that ABCA1 activity altered Hs294T spontaneous migration, we investigated more precisely how ABCA1 influenced the motility of melanoma cells. In order to migrate, cells need to interact with the ECM and form structures such as focal adhesion (FA) sites. During the maturation of FAs, the focal adhesion kinase (FAK) is recruited and autophosphorylated, which leads to the recruitment of other proteins [37]. We assessed whether the localization of the phosphorylated active form of FAK (pFAK^397^) within FAs was impacted by the ABCA1 activity. Using confocal microscopy, we observed that pFAK^397^ was localized throughout the cells but preferentially within distinguishable foci in contact with F-actin at the periphery of the scrambled and DMSO-treated cells, which corresponds to FAs structures. However, the distribution of pFAK^397^ in *ABCA1* KO and probucol-treated cells appeared more dispersed with a partial loss of defined foci seen in the control cells (Fig. 4A–D). Quantification showed that the number of cells containing pFAK^397^-rich foci was by around 50% and 40% diminished in *ABCA1* KO and probucol-treated cells, respectively, in comparison to scrambled and DMSO-treated cells (Fig. 4E and F). Interestingly, immunoblot analysis indicated a similar quantity of phosphorylated pFAK^397^ in all conditions (Fig. 4G–J). These results suggest that ABCA1 activity is not required for the phosphorylation of FAK but rather impacts the localization and stabilization of pFAK^397^ in FAs.

### ABCA1 activity impacts the distribution of active integrin β3 within the PM

**Fig. 5:**
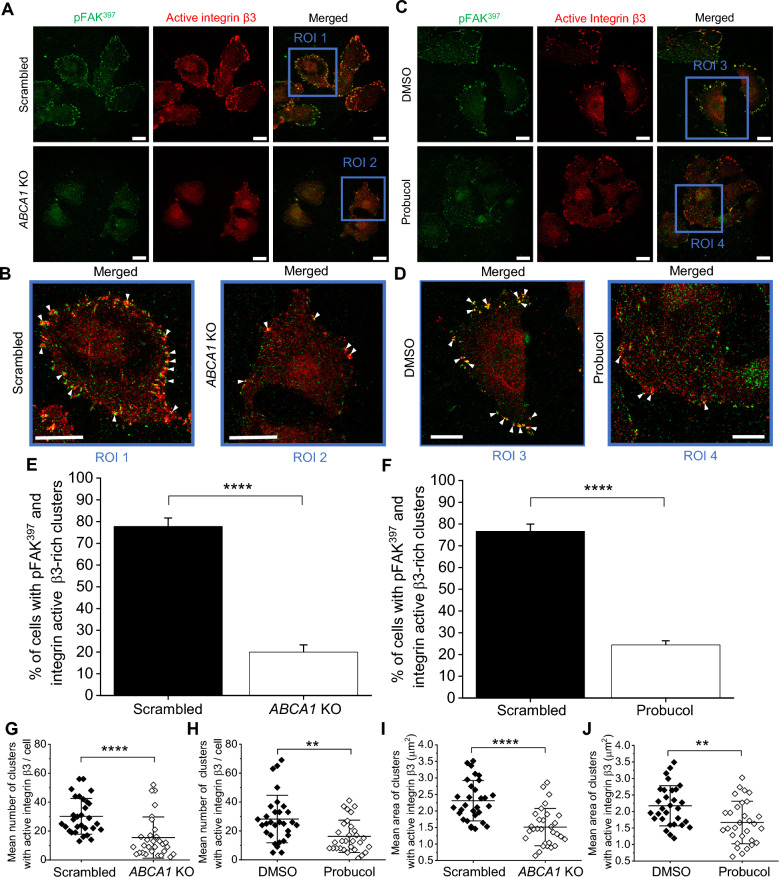
Active integrin β3 distribution and colocalization with pFAK^397^ within focal adhesions are influenced by ABCA1 activity.** A** and **C** Representative confocal images of scrambled and *ABCA1* KO cells (**A**) or DMSO- and probucol-treated cells (**C**) stained with an antibody recognizing pFAK^397^ followed by a secondary antibody conjugated with Alexa Fluor 488 (green) and antibody against active integrin β3 followed by secondary antibody conjugated with Alexa Fluor 647 (red). Scale bars represent 20 µm. **B** and **D** Magnified view of scrambled (ROI1) and *ABCA1* KO (ROI2) cells (**B**) from (**A**) or of DMSO- (ROI3) and probucol-treated (ROI4) cells (**D**) from (**C**). White arrowheads point at pFAK^397^-rich active integrin β3 clusters. Scale bars represent 20 µm. **E**,** F** Quantification of the percentage of the cells with active integrin β3 clusters enriched in pFAK^397^ of scrambled and ABCA1 KO cells (**E**) or DMSO- and probucol-treated cells (**F**) (n = 30 from three independent experiments, unpaired Student t-test). **G**, **H** Quantification of clusters number of active integrin β3 of scrambled and *ABCA1* KO cells (**G**) from (**A**) or DMSO- and probucol-treated cells (**H**) from (**C**) (n = 30 from three independent experiments, unpaired Student t-test). **I**, **J** Quantification of the clusters size of active integrin β3 of scrambled and *ABCA1* KO cells (**I**) from (**A**) or DMSO- and probucol-treated cells (**J**) from (**C**) (n = 30 from three independent experiments, unpaired Student t-test). *ROI* region of interest. Data are mean ± SD. **p ≤ 0.01 and ****p ≤ 0.0001

It has been reported that integrin β3, upon binding of the ECM ligands, recruits FAK at the forming FAs [38]. We thus investigated whether the ABCA1 depletion or inhibition, which abrogated pFAK^397^ stable recruitment to FAs was due to a defect of integrin β3 localization and function. Confocal images of cells labeled for pFAK^397^ and active integrin β3 showed that in scrambled and DMSO-treated cells, active integrin β3 was distributed across the whole cell body and was forming clusters at the perimeter of the cells that were colocalizing with pFAK^397^-rich foci. The cellular distribution of the latter was similar to previously described. Strikingly, in *ABCA1* KO and probucol-treated cells we observed a partial loss of active integrin β3 clusters within FAs, which was accompanied by the loss of colocalization with pFAK^397^ foci (Fig. 5A–F). Quantification confirmed that in *ABCA1* KO and probucol-treated cells, the active integrin β3 clusters were approximately twofold less numerous and 1.5-fold smaller compared to scrambled and DMSO-treated cells (Fig. 5G–J).

As the depletion or inhibition of ABCA1 activity did not completely abolish spontaneous migration, we investigated if the cells were still able to form mature FAs independently of integrin β3 signalization. To do so, we used immunofluorescence to analyze the studied cells labeled for active integrin β3 and for α-parvin, a protein involved in cell spreading and migration via regulation of actin cytoskeletal dynamics and association with FA complexes [39]. The confocal images showed that even though the diminished number and size of active integrin β3 clusters, *ABCA1* KO and probucol-treated cells were able to form mature FAs, as α-parvin distribution was specific to the formation of these structures (Additional file 2: Fig. S4A–D). Moreover, the α-parvin expression level was not affected by the loss of ABCA1 activity (Additional file 2: Fig. S4E, F and H, I). As the formation of FAs is involved in cell spreading on surfaces, we measured this parameter in all conditions and we did not see any differences, suggesting that ABCA1 activity is not influencing the proper localization of α-parvin within the FAs (Additional file 2: Fig. S4G and J). In conclusion, the ABCA1 activity seems to promote the activation of integrin β3 upon ECM binding that will stably recruit FAK to form fully active FAs. However, FAs were still formed via other mechanisms not influenced by the ABCA1 activity.

### ABCA1 activity modifies the PM lateral organization, order and fluidity

Integrin activation and FAK recruitment depend on the PM organization, especially for their localization in nanodomains enriched in cholesterol and phospholipids called lipid rafts [40]. We took advantage of spot-variation fluorescent correlation spectroscopy (svFCS), a highly spatio-temporally resolved microscopy technique that allows to precisely measure the diffusion parameters of specific components at the PM of living cells. [25, 41]. To assess the impact of ABCA1 on the PM of control, *ABCA1* KO and probucol-treated cells, we monitored the lateral diffusion of fluorescently labeled sphingomyelin (Bodipy-SM), a lipid probe that preferentially localizes within lipid rafts of the PM [8]. The diffusion laws (Additional file 2: Fig. S5) allowed us to conclude that under all studied conditions, the Bodipy-SM diffusion was confined in lipid rafts as the *t*_0_ value was positive. However, we observed a difference between scrambled and DMSO-treated cells with *t*_0_ of 11.48 and 11.70 ms, respectively compared to *ABCA1* KO and probucol-treated cells, with *t*_0_ of 14.30 and 14.15 ms, respectively. This suggests that in the cells with active ABCA1, there were less constraints for the Bodipy-SM diffusion within their PM and that ABCA1 is indeed able to modify the lateral organization of the PM of Hs294T cells.

**Fig. 6:**
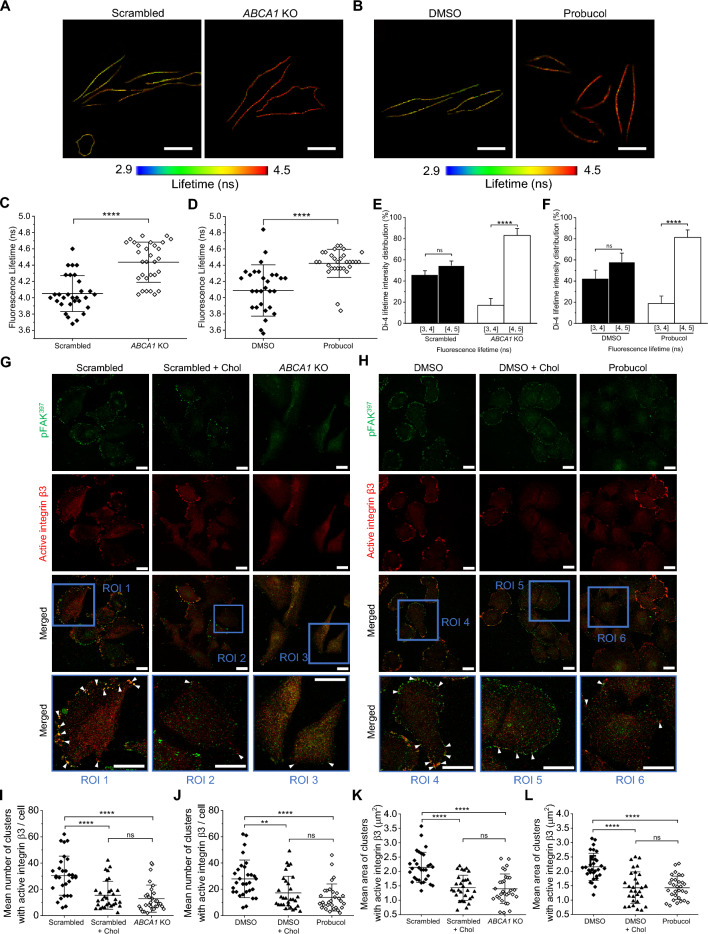
ABCA1 regulates Hs294T PM lateral organization, fluidity and order to promote the formation of fully active FAs.** A**, **B** Representative FLIM images of cellular PM, which were obtained after staining of scrambled and *ABCA1* KO cells (**A**) or DMSO- and probucol-treated cells (**B**) with the Di-4 probe. The code color scale represents the average lifetime of Di-4 with 2.9 ns as a minimum (blue) and 4.5 ns as maximum (red). Scale bars reprensent 20 µm. **C**, **D** Quantitative analysis of Di-4 mean lifetime for scrambled and *ABCA1* KO cells (**C**) and for DMSO- and probucol-treated cells (**D**) (n = 30 from three independent experiments, unpaired Student t-test). **E**, **F** Distribution represented as % of Di-4 intensity lifetime separated into two segments: Di-4 intensity with a lifetime between 3 and 4 ns [3, 4] and between 4 and 5 ns [4, 5] for scrambled and *ABCA1* KO cells (**E**) and for DMSO- and probucol-treated cells (**F**) (n = 30 from three independent experiments, unpaired Student t-tests). **G**, **H** Representative confocal images of scrambled and *ABCA1* KO cells (**G**) or DMSO- and probucol-treated (**H**) cells loaded or not with cholesterol using a methyl-β-cyclodextrin-cholesterol complex (Chol) for 30 min. Upon fixation, the cells were stained with an antibody recognizing pFAK^397^ followed by a secondary antibody conjugated with Alexa Fluor 488 (green) and an antibody against active integrin β3 followed by a secondary antibody conjugated with Alexa Fluor 647 (red). The region of interest (blue squares) of the cells was magnified (ROI). White arrowheads point at pFAK^397^-rich active integrin β3 clusters. Scale bars represent 20 µm. **I**, **J** Quantification of clusters number of active integrin β3 of scrambled and *ABCA1* KO cells (**I**) or DMSO- and probucol-treated cells (**J**) (n = 30 from three independent experiments, one-way ANOVA with Sidak’s multiple comparisons test). **K**, **L** Quantification of the size of clusters with active integrin β3 of scrambled and *ABCA1* KO cells (**K**) or DMSO- and probucol-treated cells (**L**) (n = 30 from three independent experiments, one-way ANOVA with Sidak’s multiple comparisons test). Data are mean ± SD. *ns* non-significant, *p ≤ 0.05, **p ≤ 0.01, ***p ≤ 0.001 and ****p ≤ 0.0001

To further study the effect of ABCA1 activity on the Hs294T PM organization, we performed fluorescence lifetime imaging microscopy (FLIM) on the living cells using an order sensitive probe, Di-4-ANEPPDHQ (Di-4). This dye exhibits a fluorescent lifetime shift depending on lipid order in a bilayer [42] and was already implemented by us to distinguish lipid packing within cellular PM [43, 44]. The FLIM image analysis (Fig. 6A and B) revealed a significant increase in the average lifetime of the Di-4 in *ABCA1* KO and probucol-treated cells, 4.43 and 4.42 ns, respectively, compared to scrambled and DMSO-treated cells, 4.05 and 4.09 ns, respectively, suggesting that ABCA1 activity increased the PM fluidity and loss of ABCA1 activity altered the PM order (Fig. 6C and D). Further analyses of the Di-4 fluorescence lifetime distribution between a short lifetime (between 3 and 4 ns, [3, 4]) and a long lifetime (between 4 and 5 ns, [4, 5]) regime, which represent a lower and a higher lipid packing, respectively, confirmed the implication of ABCA1 activity on the PM fluidity. Indeed, control cells exhibited a heterogenous distribution with around 45% of a short lifetime and 55% of a long lifetime, whereas ABCA1-depleted and -inactivated cells presented rather homogenous distribution with more than 80% of the signal representing a longer lifetime (Fig. 6E and F). It suggests that ABCA1 redistributes cholesterol within the PM, thus leading to the formation of PM regions of distinct lipid order and fluidity. Moreover, Di-4 has been shown to be influenced by cholesterol within the PM [45] thus providing more evidence about the PM accumulation of cholesterol when ABCA1 activity is lost. Altogether, these results indicate that ABCA1 activity impacts lateral organization of the PM of Hs294T cells by making them more fluid and that the loss of ABCA1 activity leads to cholesterol accumulation and locally changed order of the PM.

### Loading of the PM with cholesterol alters localization of active integrin β3

Previously, we showed that loading cells with methyl-β-cyclodextrin-complexed-cholesterol (Chol) can efficiently increase cholesterol content in PM and mimic a physiological cholesterol accumulation [8, 21]. Therefore, we loaded the control cells with Chol to corroborate our hypothesis that the loss of ABCA1 activity indeed decreases the PM fluidity because of cholesterol accumulation, which further leads to a defect in cell motility via pFAK^397^ and active integrin β3 cluster altered colocalization. FLIM analysis of scrambled and DMSO-treated cells loaded with cholesterol indicated that the lifetime of Di-4 and its distribution was similar to non-loaded *ABCA1* KO and probucol-treated cells (Additional file 2: Fig. S6A–D), suggesting that indeed the PM of the cells was enriched in cholesterol. We then assessed once again with confocal microscopy the subcellular localization of pFAK^397^ and active integrin β3 within these cells. Cholesterol-loaded cells were partially lacking active integrin β3 clusters, similarly to *ABCA1* KO and probucol-treated cells and that pFAK^397^ was less colocalized with the active integrin β3 clusters (Fig. 6G and H, Additional file 2: Figs. S6E and F). Moreover, quantification of active integrin β3 staining showed no significant differences in the mean number of clusters nor their size, while comparing cholesterol-loaded scrambled and DMSO-treated cells with *ABCA1* KO and probucol-treated cells (Fig. 6I–L). This suggests that indeed overaccumulation of cholesterol in the PM was altering active integrin β3 cluster formation and thus retaining pFAK^397^ at the FAs. Our results put forward for consideration that ABCA1 promotes proper focal adhesion formation and functioning by modulating PM cholesterol content and thus its lateral organization, fluidity and order.

## Discussion

**Fig. 7:**
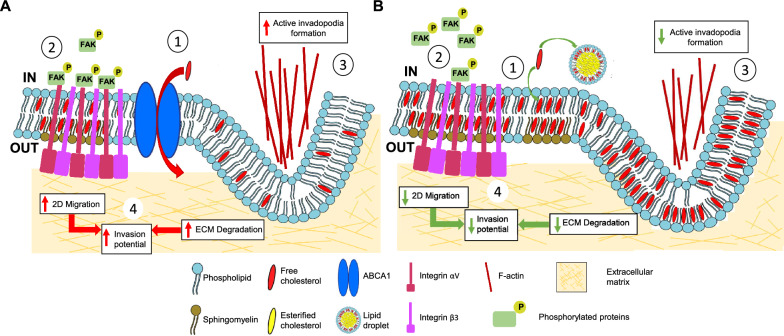
Model of the ABCA1 activity influence on Hs294T cells motility and ECM degradation processes. **A** (1) ABCA1 activity influences the cholesterol distribution within Hs294T PM, creating a heterogeneous lipid packing where lipids and membrane proteins can diffuse actively. (**2**) This specific PM lateral organization leads to the formation of integrin αVβ3 clusters that elicit activation of integrin β3 and trigger stable docking of phosphorylated FAK at the FAs and migration of the cells. (3) The specific PM lateral orsganization also influences the formation of active invadopodia which in turn increases the ECM degradation. (4) The rise in migration and the ECM digestion finally enhances the melanoma cells’ invasion potential. **B** (1) Loss of ABCA1 expression or activity triggers the accumulation of cholesterol within the PM and its excess is transformed into esterified cholesterol in lipid droplets. The PM fluidity decreases as the lipid packing rises, making the PM more homogeneous. (2) The decrease of PM fluidity destabilizes the integrin αVβ3 (with active integrin β3) clusters formation and the retaining of phosphorylated FAK at FAs, thus inhibiting the motility of the cells. (3) Accumulation of cholesterol and alteration of the PM lateral organization also inhibit the active invadopodia formation, partially preventing the ECM degradation. (4) Combined decrease in cells migration and ECM degradation lowers melanoma cells’ metastatic potential

Investigation of ABCA1 impact on cancer development has been analyzed in several studies. However, to date, ABCA1 implication in human melanoma progression has not been explored. Our first observation that high ABCA1 expression level in clinical samples correlates with more advanced tumors indicates an important role of ABCA1 in melanoma progression. Moreover, ABCA1 expression clearly correlates with a shorter cancer-specific overall survival and shorter disease-free survival. This phenomenon was already shown for colorectal cancer [12], supporting our observations. As ABCA1-mediated cholesterol transport modifies cellular plasma membrane lateral organization, the transporter activity may influence the function of membrane proteins involved in carcinogenesis. The loss of ABCA1 activity in Hs294T cells leads to an accumulation of cholesterol within the cell, which lowers their invasion and migration potential. We demonstrate that this diminution of invasion potential is related to a loss of the ECM degradation by the ABCA1-depleted or -inhibited cells and explained by an altered invadopodia formation. Moreover, in the cells that lost the ABCA1 activity, migration processes are altered by an improper activation and localization in FAs of active integrin β3 leading to an abrogated stable docking of phosphorylated FAK into the FAs. Finally, we bring the evidence that these events occur because of disruption of the PM’s cholesterol content, which leads to the alteration of the PM lateral organization, order and fluidity of the cells. Therefore, we propose a mechanism for Hs294T cells, in which ABCA1 controls the distribution of PM cholesterol content and organization, allowing the formation of active invadopodia digesting the ECM by the cells and proper stable retaining of active FAK within active integrin β3-enriched FAs leading to enhanced invasion and migration processes (Fig. 7). Altogether, our in vitro results may help to understand the poor prognosis of patients and aggressiveness of primary cutaneous melanoma with high level of ABCA1.

We showed that the loss of ABCA1 activity decreased the extent of digestion of the ECM. However, MMP-2 and MMP-9 activities were not altered in the zymography assay. It has been shown that MMPs localization within the PM was also important for ECM degradation [46] as well as recruitment of MMPs towards ECM degrading invadopodia [47]. Thus, it may suggest that it is not about the difference in the activity but rather improper localization of MMPs that stays behind the ECM degradation inhibition. Indeed, the observed reduced number of active invadopodia combined with the disruption of the PM lateral organization in *ABCA1* KO and probucol-treated cells could alter the proper localization of MMPs rather than their activity, leading to a partial abolition of ECM degradation dependent on invadopodia.

We demonstrated that partial inhibition of motility of cells without ABCA1 activity was due to a defect of pFAK^397^ proper localization towards FAs. Recruitment of FAK within the FAs and its autophosphorylation is crucial for cells migration processes to promote FAK scaffolding with specific proteins, which leads to the maturation of FAs. Furthermore, pFAK^397^ long-term residency in FAs triggers their disassembly at the cell tail enhancing their motility [48, 49]. Interestingly, we did not observe changes in the phosphorylation level of FAK. In the study on the role of nestin, a cytoskeletal intermediate filament on prostate cancer cells invasion, Hyder et al. also showed that pFAK^397^ localization, but not its level, was altered in nestin-downregulated cells [50]. The authors suggested that this event could be explained by a spatial regulation of pFAK^397^ rather than a global change in signaling since FAK function seems to be modulated locally rather than globally [51, 52]. Our observations are similar and suggest that the ABCA1 activity, by impacting the PM lateral organization of Hs294T cells, regulates pFAK^397^ stable spatial retention to FAs but not its autophosphorylation. Combined with the observed pFAK^397^ dispersed distribution throughout the cells lacking ABCA1 activity, it suggests that FAK could still be recruited to active integrin β3 clusters localized in other regions of the PM than FAs, for its autophosphorylation but could not remain in mature FAs possibly because of the lack of stabilization within the active integrin β3 clusters. FAK may also bind integrins-containing endosomes where it is phosphorylated and activated, promoting cell growth independently of their anchorage [53].

This abrogated stable retaining of active FAK within FAs was due to a decrease of active integrin β3 clusters formation in cells that lost the ABCA1 activity. An increased level of integrin β3 is closely associated with elevated invasion and metastasis potential of melanoma cells [54]. Additionally, an increased amount of integrin β3 correlates with increased rates of melanoma metastases [55]. Activation of integrin β3 upon the integrin-ECM interaction leads to the formation of active integrin clusters that are required for downstream signalization. Inhibition of integrin β3 activity in melanoma A375M cells leads to a diminution of invasion abilities [56], which is coherent with our observation that the cells lacking active integrin β3 clusters within FAs had lower invasion potential. Though we observed a diminished number of active integrin β3-rich FAs in the ABCA1-inactive cells, these cells formed α-parvin-rich FAs to the same extent as the control cells. One explanation can be extrapolated from an observation done on integrin β1-rich FAs of breast carcinoma cells [57]. It was shown that within one FA there is a mixture of active and inactive clusters of integrin β1. Apparently, here the ratio of active to inactive integrin β3 is much higher in control cells compared to the cells devoid of active ABCA1. Nevertheless, alteration in FAs functioning correlates with affected migration and invasion in the cells, which corresponds to our previous studies on melanoma cells and changes in FAs’ morphology [19, 22, 58]. Finally, it has been reported that integrin β3 expression in lung carcinoma cells is required for the ECM degradation by invadopodia [59] and that active MMP-2 colocalizes with integrin αvβ3 at the melanoma cell surface [60]. These studies help to explain the observed diminution of invadopodia formation and gelatin degradation by the cells that lost ABCA1 activity and active integrin β3 clusters formation.

Integrin β3 proper localization within the PM and subsequent signalization are influenced by PM cholesterol [61, 62]. Thus, we hypothesize that the ABCA1 activity impacts active integrin β3 clusters formation by modulating cellular PM organization. Together, svFCS and FLIM analyses brought evidence that indeed the ABCA1 activity was modulating the PM lateral organization and order. Loss of cholesterol export and redistribution within the PM lead to its decreased fluidity. This specific membrane state could then slow down the diffusion of protein within the PM and alter their clustering within nanodomains, preventing further activation and signalization. We tried to mimic this condition by overloading the PM of Hs294T cells using an MβCD: cholesterol complex and we did observe a similar repartition and size of active integrin β3 clusters in case of *ABCA1* KO, probucol-treated and cholesterol loaded cells implying that cholesterol accumulation within the PM modifies localization of proteins. Intriguingly, it has been reported that integrin αvβ3 is preferentially sequestered into liquid-ordered (l_o_) asymmetric lipid bilayers within reconstituted systems [63]. Hence, the active integrin β3 clusters could be specifically localized within asymmetric lipid rafts in the cells. Additionally to its cholesterol efflux activity, ABCA1 is also able to flop phospholipids and cholesterol from the inner leaflet toward the outer leaflet of the PM [64], thus potentially creating a favorable PM domain for active integrin β3 clusters formation and subsequent migration of the cells. Loss of ABCA1 activity could then prevent further asymmetry in this context and abrogate formation of active cluster β3 by altering the preferential sequestration in lipid rafts.

Our results suggest an altered fluidity of the cellular PM that is provoked by cholesterol accumulation rather than depletion in case of loss of the ABCA1 activity. Similarly, Zhao et al. reported that in mice injected with breast tumor cells and in human breast cancer cells, inhibition of ABCA1 activity was suppressing metastatic properties by an accumulation of cellular cholesterol content and a decreased fluidity of the cellular PM [65]. Here, we corroborate this observation in human melanoma cells. Nonetheless, we also provide more precise details of the PM lateral organization as svFCS is spatially and temporally more resolved than Fluorescence Recovery After Photobleaching (FRAP), the technique used by the authors to determine the PM fluidity, and our FLIM analysis displays precise data on lipid packing. Even though our FLIM image acquisition was rather long (between 30 s and 1 min), by combining this technique with svFCS, we managed to obtain information on the dynamic PM organization, including global and local lipid order parameters.

In conclusion, we show, to our utmost knowledge, for the first time a clinical correlation between human melanoma progression and ABCA1 expression. Moreover, we demonstrate the impact of the ABCA1 activity on human melanoma cells, and more precisely on their motility. Furthermore, we propose the following mechanism in which Hs294T melanoma cells rely on ABCA1-mediated modulation of their PM lateral organization, fluidity and order via PM cholesterol content redistribution allowing stable recruitment of pFAK^397^ towards FAs enriched in active integrin β3 clusters, thus increasing the cells motility potential. Finally, we put another brick in understanding the ABCA1 influence on melanoma cells PM organization leading to their metastatic transition and aggressiveness in the human body. The combination of our clinical and in vitro results advocates that ABCA1 might be used as a potential clinical marker for aggressive melanoma and eventually a target in anti-cancer therapies.

## Supplementary Information

**Additional file 1: Table S1.** Correlations between ABCA1 expression and clinicopathologic parameters of primary tumors in cutaneous melanoma patients.

**Additional file 2: Figure S1. **ABCA1 level is inversely proportional to cholesterol amount in melanoma cell lines. **A** Representative immunoblots of ABCA1 of WM1341D, SK-Mel-28, A375, WM9 and Hs294T cells. Vinculin was used as a loading control. **B** Quantification of ABCA1 level from. One-way ANOVA with Sidak’s multiple comparisons test was used for statistical analyses and only the statistical comparisons between Hs294T and the other cells lines are presented. **C** Total cholesterol quantitative measurement assay of WM1341D, SK-Mel-28, A375, WM9 and Hs294T cells. 5 μg of protein were used for each cell type. Data are mean ± SD. ns: non-significant, **p ≤ 0.01, ***p ≤ 0.001 and ****p ≤ 0.0001. **Figure S2. **ABCA1 activity is promoting migration and invasion ability in WM1341D and WM9 cell lines. **A**, **B **Calculated covered distances of WM1341Dor WM9DMSO- and probucol-treated cells after 72h. **C**, **D** Relative invasiveness of WM1341Dor WM9DMSO- and probucol-treated cells expressed as fold change of DMSO-treated cells after 24h. Data are mean ± SD. **p ≤ 0.01 and ****p ≤ 0.0001. **Figure S3.** ABCA1 activity does not influence MMP-2 and MMP-9 degradation abilities. **A**, **B** Representative gelatin gels of zymography assay for MMP-2 and MMP-9 activity assessment of conditioned medium from scrambled and *ABCA1* KO cellsor from DMSO- and probucol-treated cells. The same amount of samples was loaded in SDS-PAGE in parallel and gels were stained with Coomassie for total protein visualization and used as a loading control for gelatin gels. Bands on the left of Coomassie-stained gels represent the size protein ladder. **C**, **D** Quantification of the MMP-2 and MMP-9 activity expressed as % of active MMPs per Pro-MMPs of conditioned medium from scrambled and *ABCA1* KO cellsfromor of conditioned medium from DMSO- and probucol-treated cellsfrom. Data are mean ± SD. ns: non-significant. **Figure S4. **ABCA1 activity does not influence α-parvin localization and level neither cell spreading. **A**, **B** Representative confocal images of scrambled and *ABCA1* KO cellsor DMSO- and probucol-treated cellsstained with an antibody recognizing α-parvin followed by a secondary antibody conjugated with Alexa Fluor 488and antibody recognizing active integrin β3 followed by secondary antibody conjugated with Alexa Fluor 647. Scale bars represent 20 µm. **C**, **D** Magnified view of scrambledand *ABCA1* KOcellsfromor ofDMSO-and probucol-treatedcellsfrom. White arrowheads represent active integrin β3 and α-parvin-rich FAs and magenta arrowheads point at active integrin β3-poor and α-parvin-rich FAs. Scale bars represent 20 µm. **E** and **H** Representative immunoblots of α-parvin levels in scrambled and *ABCA1* KO cellsor DMSO- and probucol-treated cells. β-actin was used as a loading control. **F** and **I** Quantification of α-parvin relative level in scrambled and *ABCA1* KO cellsfromor of DMSO- and probucol-treated cellsfrom. Unpaired Student t-test were used for statistical analyses. **G** and **J** Quantification of the spreading area of scrambled and *ABCA1* KO cellsor DMSO- and probucol-treated cells. Data are mean ± SD. ns: non-significant. **Figure S5.** ABCA1 activity impacts cells’ PM lateral organization. **A**, **B** svFCS diffusion laws of fluorescently labelled sphingomyelinfor scrambledand *ABCA1* KOcellsor for DMSO-and probucol-treatedcells. **Figure S6.** Cholesterol loading disrupts cells’ PM order and pFAK^397^ stable docking to active integrin β3 clusters. **A**, **B** Quantitative analysis of Di-4 mean a lifetime of scrambled, scrambled loaded with cholesteroland *ABCA1* KO cellsor of DMSO-, DMSO loaded with cholesterol-and probucol-treated cells. **C**, **D** Distribution represented as % of Di-4 intensity lifetime separated into two segments: Di-4 intensity with a lifetime between 3 and 4 ns [3, 4] and between 4 and 5 ns [4, 5] for scrambled, scrambled loaded with cholesteroland *ABCA1* KO cellsor for DMSO-, DMSO-treated and loaded with cholesteroland probucol-treated cells. **E**, **F** Quantification from confocal imagesof the percentage of cells with active integrin β3 clusters enriched with pFAK^397^ of scrambled, scrambled loaded with cholesteroland *ABCA1* KO cellsor DMSO-, DMSO loaded with cholesterol-and probucol-treated cells. Data are mean ± SD. ns: non-significant, *p ≤ 0.05, ***p ≤ 0.001 and ****p ≤ 0.0001.

## Data Availability

This study includes no data deposited in external repositories. All data are available in the article and/or additional files and via corresponding authors upon reasonable request.

## Abbreviations

ABCA1: ATP binding cassette A1
ATP: Adenosine triphosphate
AJCC: American Joint Committee on Cancer
Chol: Methyl-β-cyclodextrin:cholesterol complex
Di-4: Di-4-ANEPPDHQ
DMEM: Dulbecco’s modified eagle medium
ECM: Extracellular matrix
EGFR: Epidermal growth factor receptor
F-actin: Filamentous actin
FA: Focal adhesion
FAK: Focal adhesion kinase
FBS: Foetal bovine serum
FLIM: Fluorescence lifetime imaging microscopy
HBSS: Hank’s balanced salt solution
HEPES: 4-(2-Hydroxyethyl)-1-piperazineethanesulfonic acid
KO: Knockout
MPP: Matrix metalloproteinase
PBS: Phosphate buffer saline
PM: Plasma membrane
SD: Standard deviation
SM: Sphingomyelin
svFCS: Spot-variation fluorescence correlation spectroscopy
WT: Wild type
