# Supplementary material for: ABCA1 transporter promotes the motility of human melanoma cells by modulating their plasma membrane organization
Source: Biol Res. 2023 Jun 13;56:32. doi: 10.1186/s40659-023-00443-4 (PMC10262546; doi:10.1186/s40659-023-00443-4)
Supplement: Supplementary file 1 — Additional file 1: Table S1. Correlations between ABCA1 expression and clinicopathologic parameters of primary tumors in cutaneous melanoma patients. [file 40659_2023_443_MOESM1_ESM.pdf]

Table S1. Correlations between ABCA1 expression and clinicopathologic parameters of primary tumors in cutaneous melanoma patients.

	ABCA1 Expression		
	**Low (H-score ≤ 200) (N = 63)**	**High (H-score > 200) (N = 47)**	**p-Value**
**Age (18−86 years) a**			0.3638
≤65	35 (56%)	22 (47%)	
>65	28 (44%)	25 (53%)	
**Gender b**			0.7001
Female	30 (48%)	25 (53%)	
Male	33 (52%)	22 (47%)	
**Primary tumor location a**			0.1644
Head/neck	3 (5%)	8 (17%)	
Extremities	29 (46%)	16 (34%)	
Trunk	29 (46%)	22 (47%)	
Hand/foot	2 (3%)	1 (2%)	
**Primary tumor (pT) c**			**<0.001**
pT1	23 (37%)	2 (4%)	
pT2	14 (22%)	5 (11%)	
pT3	13 (21%)	12 (26%)	
pT4	13 (21%)	28 (60%)	
**Sentinel lymph node biopsy status (SNLB) b**			0.3883
No metastases (SNLB−)	18 (67%)	12 (52%)	
Metastases present (SNLB+)	9 (33%)	11 (48%)	
**Regional lymph nodes status (pN) b**			0.1784
Metastases absent (pN−)	51 (81%)	32 (68%)	
Metastases present (pN+)	12 (19%)	15 (32%)	
**Distant metastases (pM) b**			0.7555
No metastases (pM−)	56 (89%)	43 (91%)	
Metastases present (pM+)	7 (11%)	4 (9%)	
**AJCC (8th edition) stage c**			**<0.001**
**I**	32 (51%)	6 (13%)	
**II**	17 (27%)	23 (49%)	
**III**	7 (11%)	14 (30%)	
**IV**	7 (11%)	4 (9%)	
**Recurrence b**			**0.003**
No	48 (76%)	22 (47%)	
Yes	15 (24%)	25 (53%)	
**Breslow thickness c**			**<0.001**
≤1 mm	23 (37%)	2 (4%)	
1.01−2.00 mm	13 (21%)	5 (11%)	
2.01−4.00 mm	14 (22%)	12 (26%)	
>4 mm	13 (21%)	28 (60%)	
**Clark level c**			**0.001**
I	0 (0%)	0 (0%)	
II	26 (41%)	3 (6%)	
III	17 (27%)	22 (47%)	
IV	16 (25%)	15 (32%)	

V	4 (6%)	7 (15%)	
**Histological type a**			**<0.001**
Superficial spreading melanoma	38 (60%)	10 (21%)	
Nodular melanoma	23 (37%)	36 (77%)	
Acral lentiginous melanoma	2 (3%)	1 (2%)	
**Mitotic rate c**			**<0.001**
0	24 (38%)	0 (0%)	
1–2	10 (16%)	7 (15%)	
>2	29 (46%)	40 (85%)	
**Ulceration a**			**<0.001**
No	46 (73%)	17 (35%)	
Yes	17 (27%)	30 (64%)	
**Lymphangioinvasion a**			0.4242
No	61 (97%)	44 (94%)	
Yes	2 (3%)	3 (6%)	
**Tumor-infiltrating lymphocytes a**			**0.004**
No	6 (10%)	1 (2%)	
Non-brisk	30 (48%)	37 (79%)	
Brisk	27 (43%)	9 (19%)	
**Microsatellitosis a**			0.7645
No	61 (97%)	45 (96%)	
Yes	2 (3%)	2 (4%)	
**Regression a**			0.4242
No	61 (97%)	44 (94%)	
Yes	2 (3%)	3 (6%)	

a - chi-square test
b - Fisher exact test
c - Wilcoxon two sample test
