# Supplementary material for: ABCA1 transporter promotes the motility of human melanoma cells by modulating their plasma membrane organization
Source: Biol Res. 2023 Jun 13;56:32. doi: 10.1186/s40659-023-00443-4 (PMC10262546; doi:10.1186/s40659-023-00443-4)
Supplement: Supplementary file 2 — Additional file 2: Figure S1. ABCA1 level is inversely proportional to cholesterol amount in melanoma cell lines. A Representative immunoblots of ABCA1 of WM1341D, SK-Mel-28, A375, WM9 and Hs294T cells. Vinculin was used as a loading control. B Quantification of ABCA1 level from. One-way ANOVA with Sidak’s multiple comparisons test was used for statistical analyses and only the statistical comparisons between Hs294T and the other cells lines are presented. C Total cholesterol quantitative measurement assay of WM1341D, SK-Mel-28, A375, WM9 and Hs294T cells. 5 μg of protein were used for each cell type. Data are mean ± SD. ns: non-significant, **p ≤ 0.01, ***p ≤ 0.001 and ****p ≤ 0.0001. Figure S2. ABCA1 activity is promoting migration and invasion ability in WM1341D and WM9 cell lines. A, B Calculated covered distances of WM1341Dor WM9DMSO- and probucol-treated cells after 72h. C, D Relative invasiveness of WM1341Dor WM9DMSO- and probucol-treated cells expressed as fold change of DMSO-treated cells after 24h. Data are mean ± SD. **p ≤ 0.01 and ****p ≤ 0.0001. Figure S3. ABCA1 activity does not influence MMP-2 and MMP-9 degradation abilities. A, B Representative gelatin gels of zymography assay for MMP-2 and MMP-9 activity assessment of conditioned medium from scrambled and ABCA1 KO cellsor from DMSO- and probucol-treated cells. The same amount of samples was loaded in SDS-PAGE in parallel and gels were stained with Coomassie for total protein visualization and used as a loading control for gelatin gels. Bands on the left of Coomassie-stained gels represent the size protein ladder. C, D Quantification of the MMP-2 and MMP-9 activity expressed as % of active MMPs per Pro-MMPs of conditioned medium from scrambled and ABCA1 KO cellsfromor of conditioned medium from DMSO- and probucol-treated cellsfrom. Data are mean ± SD. ns: non-significant. Figure S4. ABCA1 activity does not influence α-parvin localization and level neither cell spreading. A, B Representative confocal [file 40659_2023_443_MOESM2_ESM.pdf]

ABCA1 transporter promotes the motility of human melanoma cells by modulating their plasma membrane organization

Ambroise Wu[1], Ewa Mazurkiewicz[2], Piotr Donizy[3], Krzysztof Kotowski[3], Małgorzata Pieniazek[4], Antonina J. Mazur[2]*, Aleksander Czogalla[1]*, Tomasz Trombik[5,6]*

[1] Department of Cytobiochemistry, Faculty of Biotechnology, University of Wrocław, Joliot-Curie 14a, 50-383 Wrocław, Poland
[2] Department of Cell Pathology, Faculty of Biotechnology, University of Wrocław, Joliot-Curie 14a, 50-383 Wrocław, Poland
[3] Department of Clinical and Experimental Pathology, Wrocław Medical University, Borowska 213, 50-556 Wroclaw, Poland
[4] Department of Oncology and Division of Surgical Oncology, Wroclaw Medical University, pl. Hirszfelda 12, 53-413 Wroclaw, Poland
[5] Department of Biophysics, Faculty of Biotechnology, University of Wrocław, Joliot-Curie 14a, 50-383 Wrocław, Poland
[6] Present address: The Chair and Department of Biochemistry and Molecular Biology, Medical University, Chodzki 1, 20-093 Lublin, Poland
* Corresponding authors : tomasztrombik@umlub.pl, aleksander.czogalla@uwr.edu.pl, antonina.mazur@uwr.edu.pl

Additional file Figures 1-6

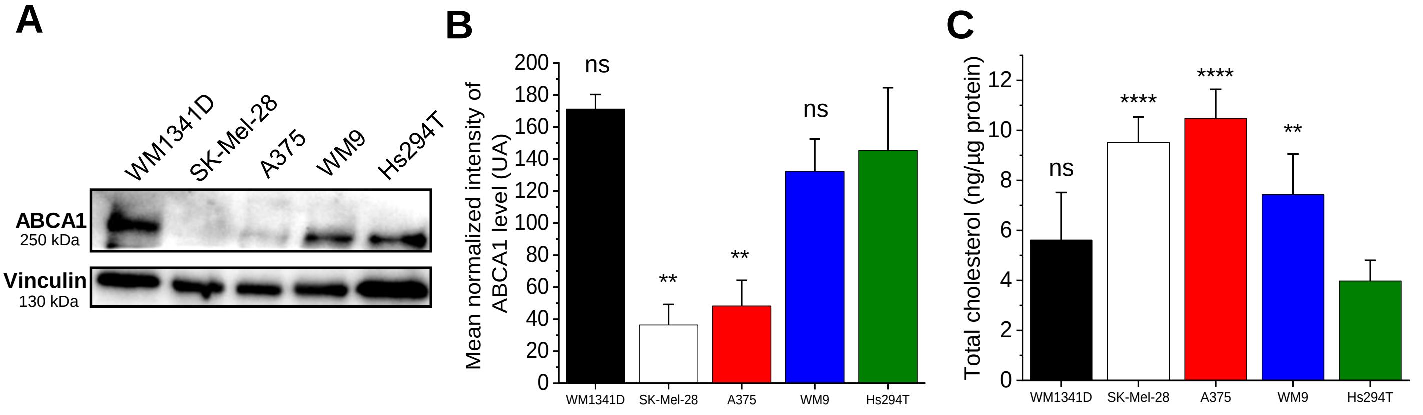
A
WM1341D
SK-Mel-28
A375
WM9
Hs294T
ABCA1
250 kDa
Vinculin
130 kDa
B
Mean normalized intensity of ABCA1 level (UA)
ns
**
**
ns
WM1341D
SK-Mel-28
A375
WM9
Hs294T
C
Total cholesterol (ng/µg protein)
ns
****
****
**
WM1341D
SK-Mel-28
A375
WM9
Hs294T

Figure S1

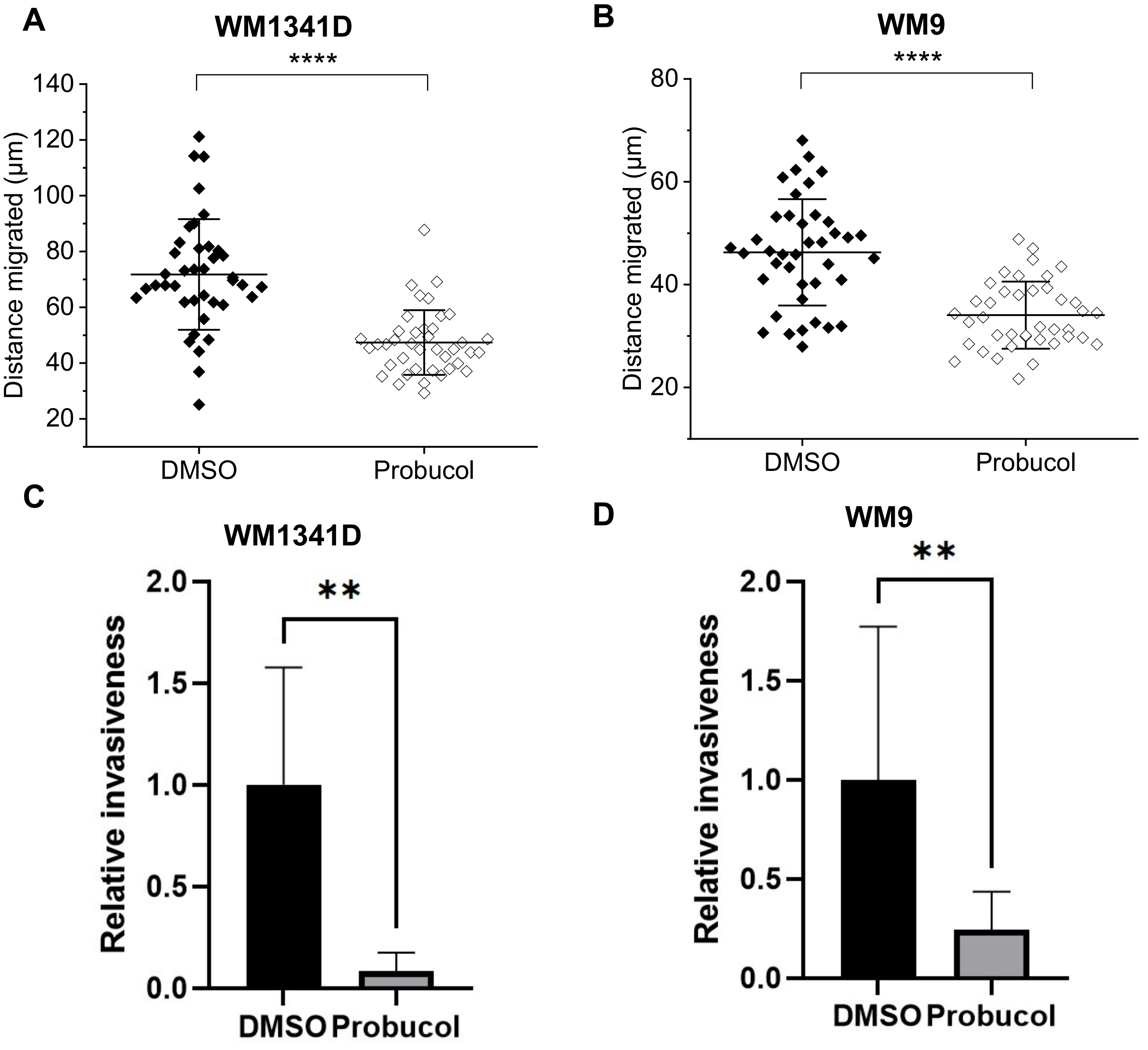
A
WM1341D
****
Distance migrated (µm)
140
120
100
80
60
40
20
DMSO
Probucol
B
WM9
****
Distance migrated (µm)
80
60
40
20
DMSO
Probucol
C
WM1341D
**
Relative invasiveness
2.0
1.5
1.0
0.5
0.0
DMSO
Probucol
D
WM9
**
Relative invasiveness
2.0
1.5
1.0
0.5
0.0
DMSO
Probucol

Figure S2

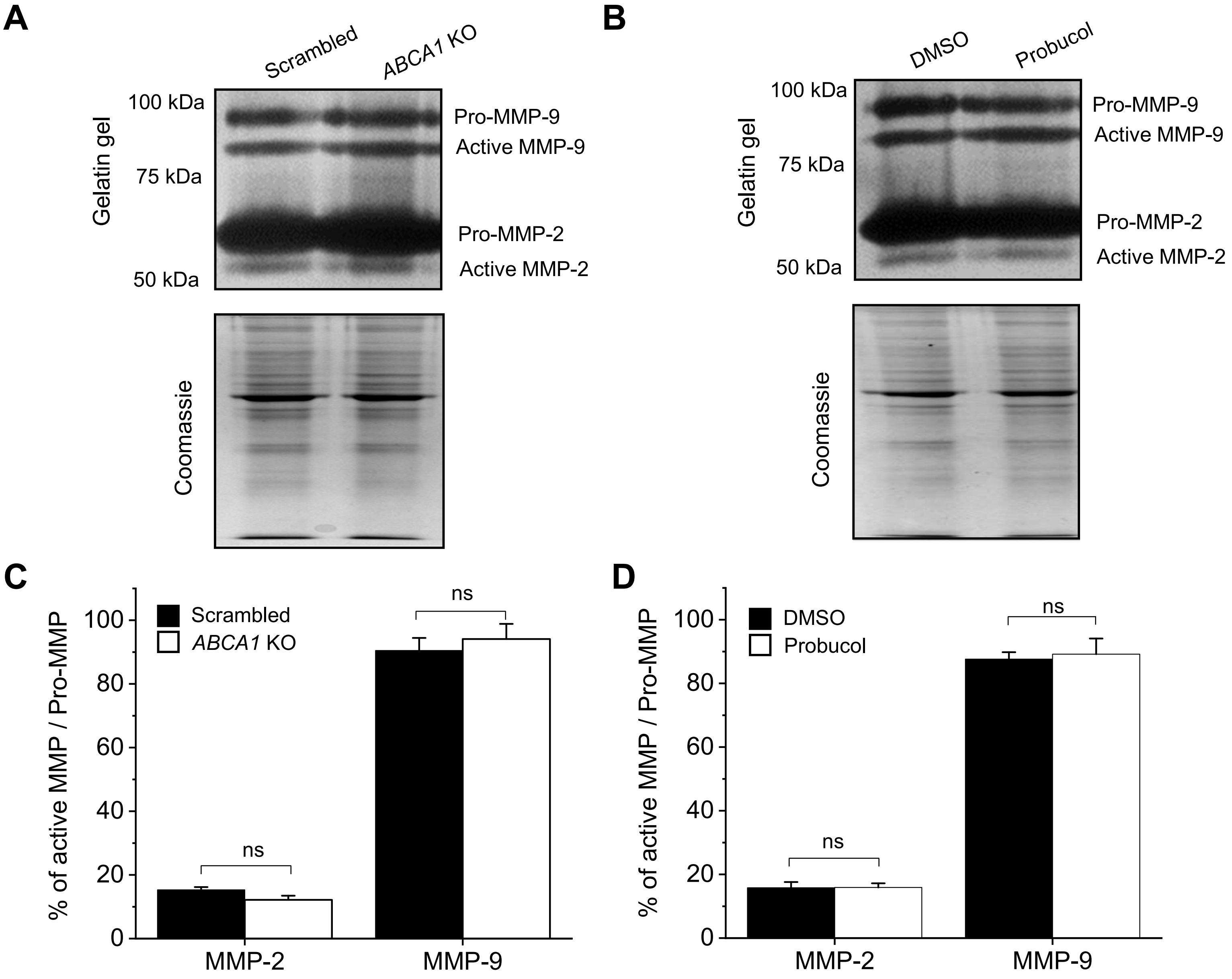

A
Scrambled
ABCA1 KO
100 kDa
75 kDa
50 kDa
Gelatin gel
Pro-MMP-9
Active MMP-9
Pro-MMP-2
Active MMP-2
Coomassie
B
DMSO
Probucol
100 kDa
75 kDa
50 kDa
Gelatin gel
Pro-MMP-9
Active MMP-9
Pro-MMP-2
Active MMP-2
Coomassie
C
% of active MMP / Pro-MMP
Scrambled
ABCA1 KO
ns
ns
0
20
40
60
80
100
MMP-2
MMP-9
D
% of active MMP / Pro-MMP
DMSO
Probucol
ns
ns
0
20
40
60
80
100
MMP-2
MMP-9

Figure S3

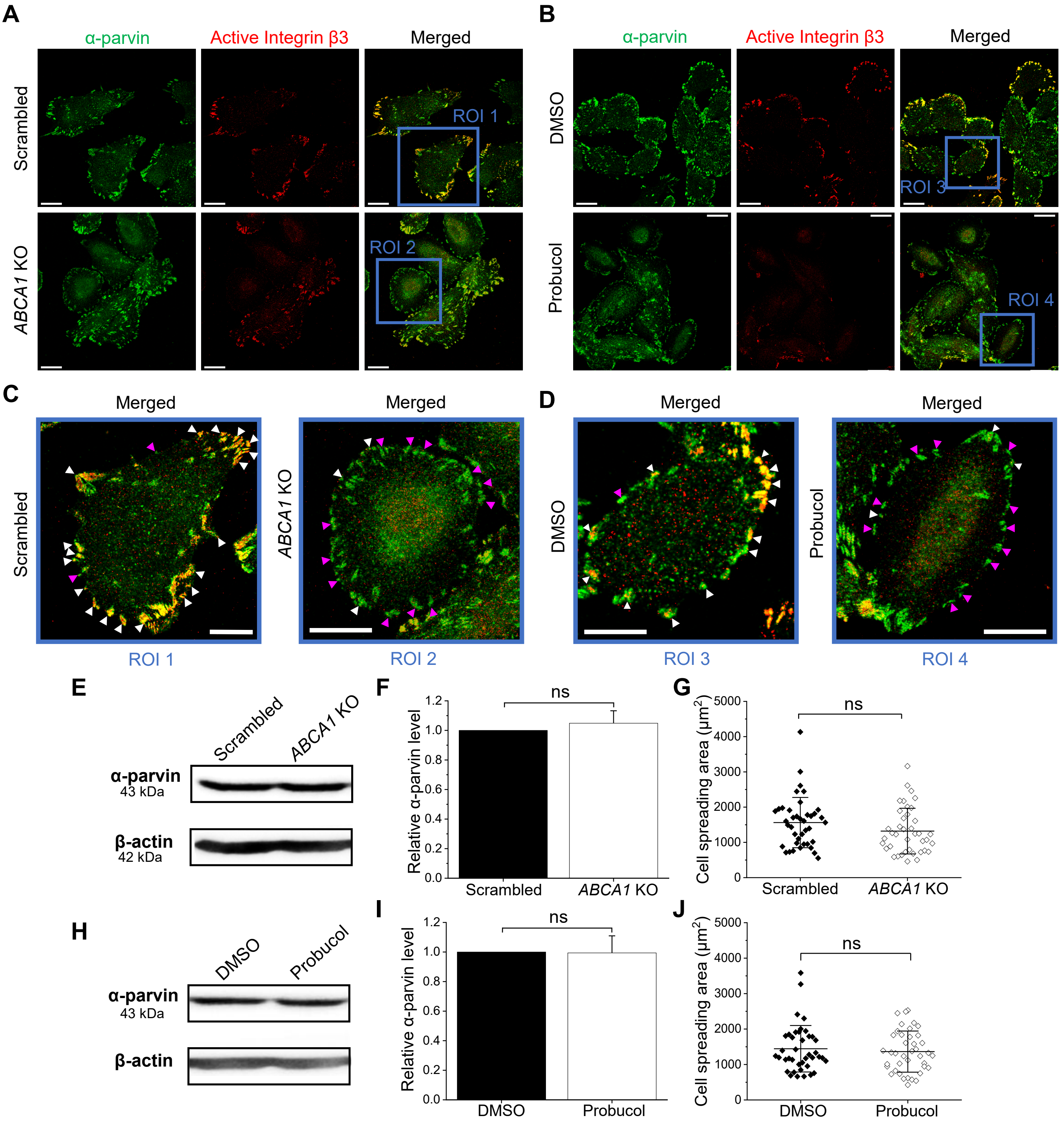
A
α-parvin
Active Integrin β3
Merged
Scrambled
ABCA1 KO
ROI 1
ROI 2
B
DMSO
Probucol
ROI 3
ROI 4
C
D
E
Scrambled
ABCA1 KO
α-parvin
43 kDa
β-actin
42 kDa
F
ns
Relative α-parvin level
G
Cell spreading area (μm²)
H
DMSO
Probucol
I
J

Figure S4

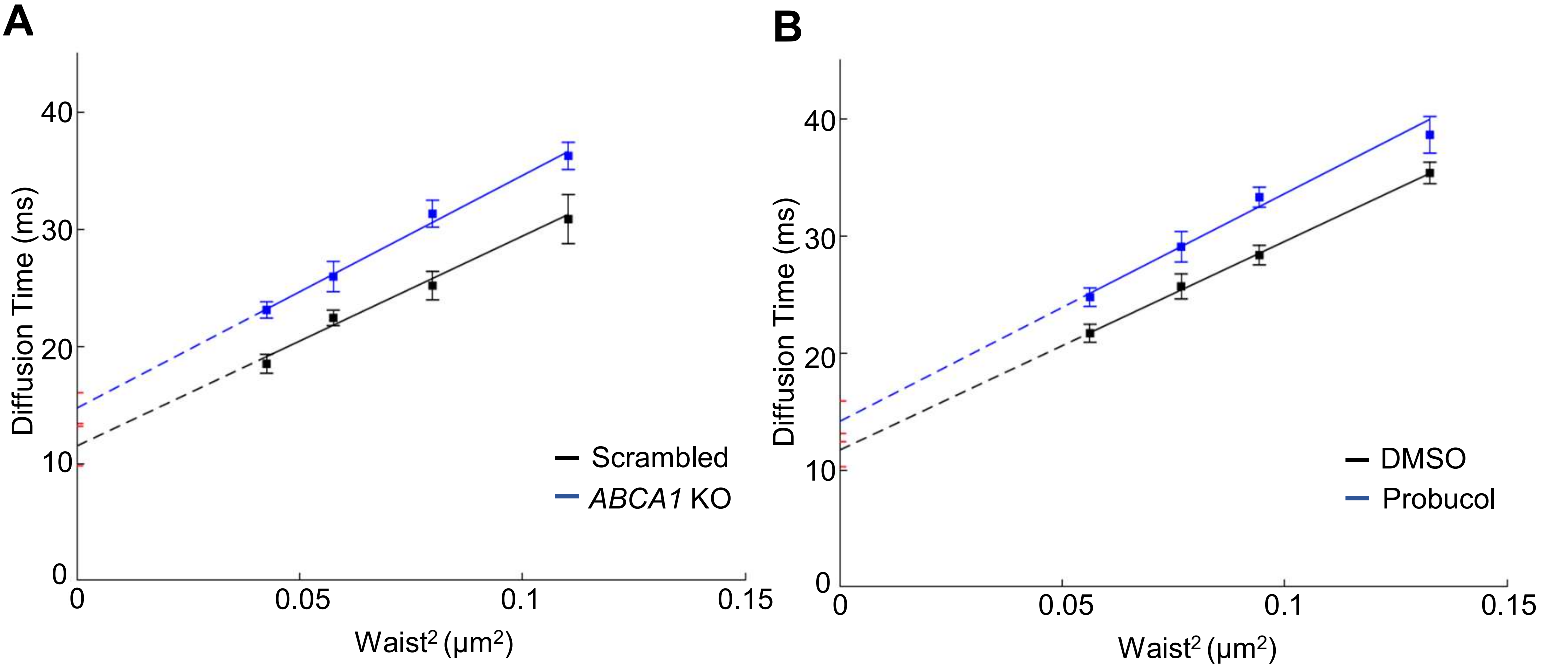
A
Diffusion Time (ms)
Waist² (μm²)
Scrambled
ABCA1 KO
B
Diffusion Time (ms)
Waist² (μm²)
DMSO
Probucol

Figure S5

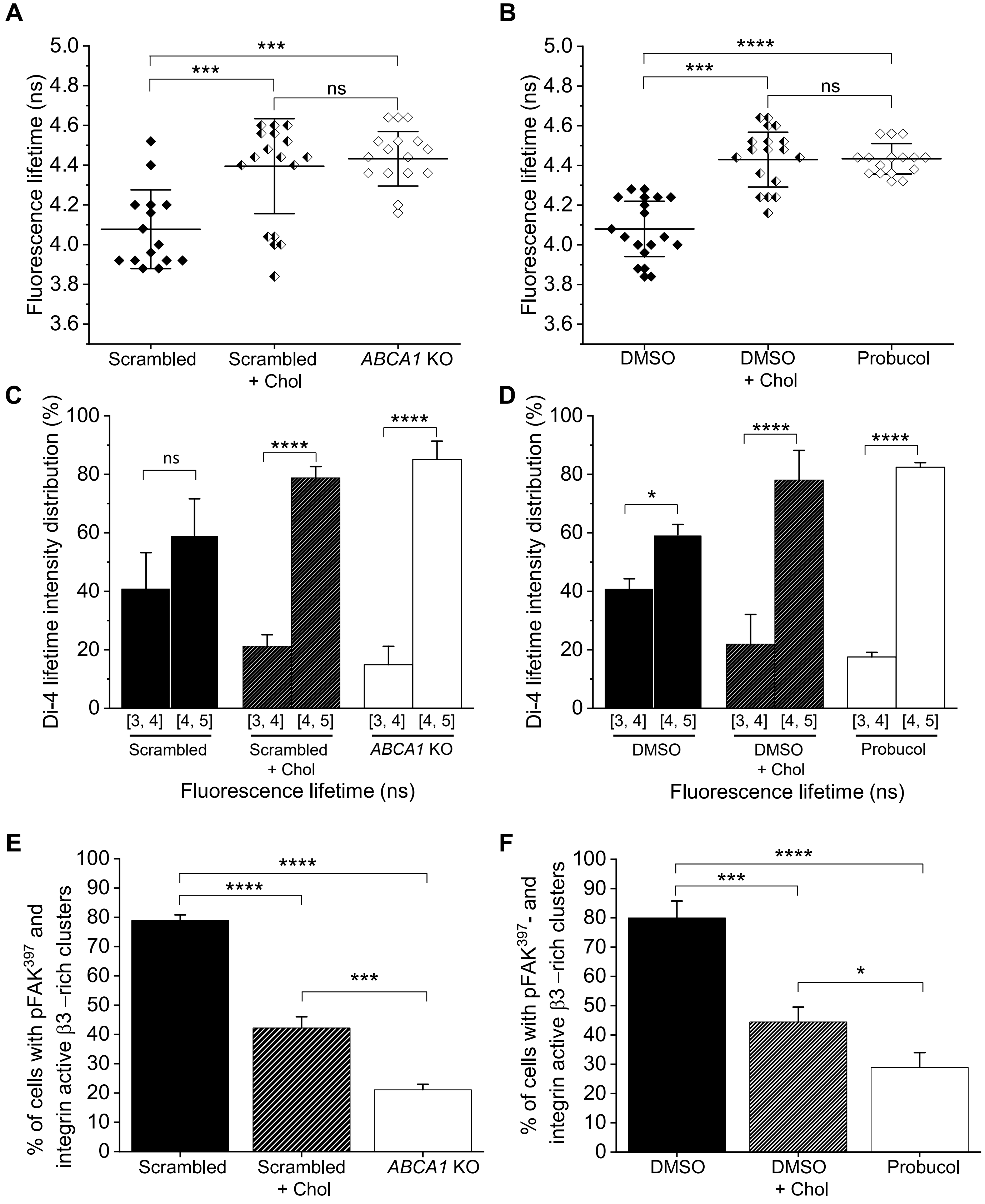
A
B
C
D
E
F
Fluorescence lifetime (ns)
Scrambled
Scrambled + Chol
ABCA1 KO
DMSO
DMSO + Chol
Probucol
***
****
ns
*
Di-4 lifetime intensity distribution (%)
[3, 4]
[4, 5]
% of cells with pFAK397 and integrin active β3 –rich clusters
% of cells with pFAK397- and integrin active β3 –rich clusters

Figure S6
